# Why? – Successful *Pseudomonas aeruginosa* clones with a focus on clone C

**DOI:** 10.1093/femsre/fuaa029

**Published:** 2020-09-29

**Authors:** Changhan Lee, Jens Klockgether, Sebastian Fischer, Janja Trcek, Burkhard Tümmler, Ute Römling

**Affiliations:** Department of Microbiology, Tumor and Cell Biology, Biomedicum C8, Karolinska Institutet, SE-171 77 Stockholm, Sweden; Clinic for Paediatric Pneumology, Allergology and Neonatology, Clinical Research Group ‘Pseudomonas Genomics’, Hannover Medical School, D-30625 Hannover, Germany; Clinic for Paediatric Pneumology, Allergology and Neonatology, Clinical Research Group ‘Pseudomonas Genomics’, Hannover Medical School, D-30625 Hannover, Germany; Faculty of Natural Sciences and Mathematics, Department of Biology, University of Maribor, Maribor, 2000, Slovenia; Clinic for Paediatric Pneumology, Allergology and Neonatology, Clinical Research Group ‘Pseudomonas Genomics’, Hannover Medical School, D-30625 Hannover, Germany; Department of Microbiology, Tumor and Cell Biology, Biomedicum C8, Karolinska Institutet, SE-171 77 Stockholm, Sweden

**Keywords:** disaggregase, FtsH, genomic island, protein homeostasis, pulsed field gel electrophoresis, whole genome sequencing

## Abstract

The environmental species *Pseudomonas aeruginosa* thrives in a variety of habitats. Within the epidemic population structure of *P. aeruginosa*, occassionally highly successful clones that are equally capable to succeed in the environment and the human host arise. Framed by a highly conserved core genome, individual members of successful clones are characterized by a high variability in their accessory genome. The abundance of successful clones might be funded in specific features of the core genome or, although not mutually exclusive, in the variability of the accessory genome. In clone C, one of the most predominant clones, the plasmid pKLC102 and the PACGI-1 genomic island are two ubiquitous accessory genetic elements. The conserved transmissible locus of protein quality control (TLPQC) at the border of PACGI-1 is a unique horizontally transferred compository element, which codes predominantly for stress-related cargo gene products such as involved in protein homeostasis. As a hallmark, most TLPQC xenologues possess a core genome equivalent. With elevated temperature tolerance as a characteristic of clone C strains, the unique *P. aeruginosa* and clone C specific disaggregase ClpG is a major contributor to tolerance. As other successful clones, such as PA14, do not encode the TLPQC locus, ubiquitous denominators of success, if existing, need to be identified.

## Teaser

Molecular epidemiology of *Pseudomonas aeruginosa* unraveled the occurence of few predominant clones that can thrive in various habitats with clone C one of the most abundant group of closely related strains that harbor common and strain specific entities which provide unique features such as xenologues of core genome genes involved in protein homeostasis to clone C strains.

## INTRODUCTION

*Pseudomonas aeruginosa* is the prototype of an environmental bacterium the adaptability of which promotes selected fractions of the population to successfully occupy anthropized environments. To understand the genetic and physiological basis of the success of abundant clones, group of closely related strains, that thrive in environmental and clinical habitats, in contrast to less abundant clones with more restricted ecological niches, is of particular interest for population genetics. To unravel the genetic basis of ubiquity, adaptability and persistence of clones and its individual members is not only highly relevant from a basic science point of view such as to unravel the impact of individuality in a successful population, but also from a clinical point of view in order to prevent the emergence and spread of multidrug resistant clones. In this review, we describe the ecological and molecular characteristics of abundant *P. aeruginosa* clone C first consciously isolated from natural and clinical habitats in Germany and Canada in the 1980s. Unravelling in more detail the genetic background and physiology of this clone, not known to extensively bearing antimicrobial resistance markers, will shed light on survival strategies of microbial organisms.

### The species *Pseudomonas aeruginosa*

The Gram-negative bacterium *Pseudomonas aeruginosa* is the type species of the genus *Pseudomonas* which consists today of almost 200 species (http://www.bacterio.net/-pseudomonas.html). With one polar flagellum, *Pseudomonas aeruginosa*, isolated the first time in 1882, was described and named by the botanist Walter Emil Friedrich August Migula (Migula 1894). The metabolic versatility and minimal growth requirements of *P. aeruginosa* characteristic for the species of the genus *Pseudomonas* in combination with robust isolation has led to the conventional view that *P. aeruginosa* is ubiquitous in nature accumulating preferentially in human-contaminated environments. Indeed *P. aeruginosa* is regularly isolated from oil-contaminated fields and sewage, but also swimming pools and household sinks (Grobe, Wingender and Truper 1995; Pirnay *et al*. 2005; Das and Mukherjee 2007). Recovery from distilled water and disinfectants such as triclosan contribute to its presence in the clinic (Lanini *et al*. 2011). *Pseudomonasaeruginosa* occurs in natural environments as diverse as natural freshwater water, the marine environment, plants, mushrooms and soil (Ojima *et al*. 2002; Khan *et al*. 2007; Kidd *et al*. 2012; Rutherford *et al*. 2018; Schroth *et al*. 2018). Due to unique products and its metabolic versatility *P. aeruginosa* also has gained interest to be used in biotechnological applications (Reetz and Jaeger 1998; Fenibo *et al*. 2019).

With eukaryotic hosts, *P. aeruginosa* shows a broad spectrum of interactions. In plants, the effect of the organism spans from growth promoting to being a plant pathogen (Rahme *et al*. 1995; Adesemoye, Obini and Ugoji 2008). Association of *P. aeruginosa* with an immunocompetent human being is usually infrequent and temporary with gastrointestinal and skin colonization (Cooke *et al*. 1970; Silvestre and Betlloch 1999; Dossel *et al*. 2012; Garcia *et al*. 2018). Approximately 5% of gastrointestinal carriage in humans points to an acquisition by produce or water in combination with an efficient colonization resistance by the gastrointestinal microbiome (Kerckhoffs *et al*. 2011). Superficial skin (hot tub folliculitis) and ear (otitis externa, also called swimmer's ear) infections with *P. aeruginosa* can be readily acquired in natural or anthropized environments with a high number of the organism (Ratnam *et al*. 1986; Ahlen, Mandal and Iversen 2001).

Upon the introduction of antibiotics, due to the innate and acquired resistance against antibiotics in combination with its nutritional minimalism, *P. aeruginosa* has developed into one of the most frequently hospital acquired (nosocomial) pathogens (Gould and Wise 1985). A local or systemic impairment of the innate or adaptive immune response such as lack of skin as an innate immune barrier in severe burn wounds, depletion of neutrophils in neutropenia, debiliated mucociliary clearance in cystic fibrosis and immune aging due to old age is usually the basis for the establishment of a successful infection with *P. aeruginosa*. As such, in a wide spectrum of infections including diabetic foot ulcer and ear infection *P. aeruginosa* is a frequent causative agent (Hatipoglu *et al*. 2014). With its notorious ability to form biofilms, *P. aeruginosa* infections are promoted by its colonization on artificial devices. Thus the prevalence of *P. aeruginosa* infections is especially prominent in catheter-associated urinary tract infection and ventilator associated pneumonia (VAP), two of the most common nosocomial acquired infection, but also in contact-lens associated keratitis in immunocompetent individuals (http://www.-antimicrobe.org/b112.asp; (Bouza *et al*. 2001; Chastre and Fagon 2002; Rello *et al*. 2006; Bjerklund Johansen *et al*. 2007; Willcox 2012)).

Prior to the introduction of genome-wide molecular techniques, the versatility of the genome, the broad spectrum of habitats and infections and the absence of unique characteristics such as virulence factors or serotypes associated with pathogenicity limited the epidemiology of *P. aeruginosa* allowing only a low discriminatory and inconclusive classification of isolates (Tümmler *et al*. 1991; Kidd *et al*. 2012; Parkins, Somayaji and Waters 2018). Furthermore, the molecular mechanisms of the environmental species *P. aeruginosa* to conduct this broad range of environmental, saphrophytic and clinical interactions remained enigmatic. The recent initiatives of genome wide typing approaches of large strain collections including whole genome sequencing, in combination with in depth investigations on the molecular analysis of gene products, begin to unravel the molecular and physiological details of such a versatility on the population and individual strain level.

### Genotyping of *Pseudomonas aeruginosa* strains

The classification of bacterial isolates on the strain level is relevant for ecology, epidemiology, taxonomy and biotechnology. Highly discriminatory genotyping methods for *P. aeruginosa* are either based on anonymous fingerprinting techniques like macrorestriction fragment pattern analysis or sequence-based typing approaches by multilocus sequence typing (MLST) and microarrays. Macrorestriction fragment pattern analysis has been made possible by the discovery of Schwartz and Cantor to separate kbp and Mbp long linear DNA fragments according to size in a gel matrix upon application of an alternately pulsed electric field (Schwartz and Cantor 1984). Generating barcode-like whole genome fingerprints created by rare cutting restriction enzymes such as SpeI is globally applicable to bacteria and hence is still the reference method for strain typing.

On the other hand, the *P. aeruginosa* MLST scheme utilizes nucleotide sequence data of internal fragments of seven housekeeping genes (https://pubmlst.org/paeruginosa/) (Kiewitz and Tummler 2000; Jolley, Bray and Maiden 2018) to scan the genetic diversity of the core genome by amplicon sequencing under high throughput. As a further development, a robust and rapid oligonucleotide microarray can type *P. aeruginosa* strains in both the conserved core and the flexible accessory genome (Wiehlmann *et al*. 2007). The microarray, hybridzed with the strain's DNA yields an electronically portable binary multimarker genotype that represents the core genome by single nucleotide polymorphisms (SNPs) and the accessory genome by markers of genomic islets and islands. A hexadecimal code summarizing the SNP genotypes assigns the strains to a clonal complex. Multimarker genotypes of 1448 strains are publicly available (Wiehlmann, Cramer and Tümmler 2015).

Examination of more than 550 *P. aeruginosa* isolates from environmental and clinical habitats by their macrorestriction SpeI fingerprints in the early 1990s identified more than 20% of the strains from various spatially and temporally separated habitats mainly from Germany to be variants of one major clone that since then is called clone C (Römling *et al*. 1994a,b). The hexadecimal code for clone C isolates reads C40A which matches in the MLST database with two rare (ST2691, ST2894) and two frequent MLST subtypes (ST17, ST845) the latter two accounting for more than 95% of clone C isolates.

### Population biology of *Pseudomonas aeruginosa*

Cumulatively, recent whole genome sequencing projects have demonstrated that the population of the cosmopolitan *P. aeruginosa* grossly consists of one ExoS-positive and one ExoU-positive clade and three small groups of distant outliers (Stewart *et al*. 2014; Hilker *et al*. 2015; Freschi *et al*. 2019). Linkage groups, consecutive genes without recombination events, are just a few hundred base pairs in size indicating gene flow by recombination between clonal complexes (Dettman, Rodrigue and Kassen 2014; Hilker *et al*. 2015). These data support the conclusion of *P. aeruginosa* to mainly exhibit a non-clonal epidemic structure as previously drawn from polyphasic data sets (Pirnay *et al*. 2009).

To unravel the population structure of *P. aeruginosa* on the level of clones, several thousand isolates from more than 1500 independent habitats of diverse geographic origin have been investigated by microarray genotyping (Wiehlmann, Cramer and Tümmler 2015). This genotyping approach identified 323 different clone types. 109 clones made up for 82% of the population, whereby the 12 or 26 most frequent clones had absolute shares of 33.4% or 50%, respectively. On the other hand, 167 and 47 clone representatives were only found once or twice, respectively. In other words, the *P. aeruginosa* population is dominated by few epidemic clonal complexes (De Soyza *et al*. 2013). Overall, the most abundant genotype was the ExoS-positive clone C (C40A) (Römling *et al*. 1994a,b; Römling *et al*. 2005). Clone C was the most abundant genotype among the isolates from chronic human infections, the second most frequent clone in acute human and animal infections and the fourth most frequent clone among the isolates from the inanimate environment, i.e. soil and aquatic habitats (Wiehlmann, Cramer and Tümmler 2015).

The *P. aeruginosa* community is more diverse in its clonal composition in soil and aquatic habitats than in the infected human host. Some *P. aeruginosa* clones like 149A, 081A or CBA3 (see Table 1) are common in the environment, but are rare or absent as causative agents of infections and thus behave like strains from the related *Pseudomonas putida/Pseudomonas fluorescens* group that are non-pathogenic for immunocompetent humans. The genetic repertoire to establish a niche in the mammalian host and/or to combat the host defense must be impaired in those clones. Consequently, in disease habitats the *P. aeruginosa* population narrows to proficient clones, which can colonize and persist in an animate host and thus, besides some generalists such as clone C (hexadecimal code C40A, Table 1) and PA14 (code D421), selects for minor clones to become dominant members of the populations in such atypical niches. A particular case is the lungs of cystic fibrosis patients (CF) where the microorganisms can be decade-long exposed to a hostile immune system and regular antimicrobial chemotherapy. These findings, made possible due to high resolution typing techniques and large strain collections, challenge the long standing dogma of environmental and clinical *P. aeruginosa* isolates being indistinguishable in their genetic properties and virulence factors.

**Table 1.** Prevalence of the 15 most common environmental *P. aeruginosa* clones in human infections.

	Relative abundance [%]^b^	
Clone^a^	Environment	Human infections
EA0A	6.5	1.5
B420	6.3	1.5
C40A	5.1	7.4
0812	4.4	1.6
F46A	4.0	0.6
E429	3.5	2.2
F429	3.3	2.1
0C2E	2.6	3.8
D421	2.1	4.3
EC2A	2.1	1.0
149A	1.9	< 0.1
081A	1.9	< 0.1
CBA3	1.9	nd^c^
4C1A	1.6	< 0.1
6E1A	1.4	nd^c^

a The clones are designated by hexadecimal code derived from a multi-marker array (Wiehlmann *et al*. 2007).

b Data refer to 1677 singular *P. aeruginosa* isolates from independent habitats purified from a collection of 3070 genotyped isolates.

c not detected

In summary, the inanimate aquatic habitats harbor the largest pool of clones out of which subgroups spread to more specialized niches. The successful colonizers are either generalists like clone C found everywhere or minor clones that are endowed with clone-specific features to adapt to this peculiar niche. Clonal fitness is thereby subject to continuous genome evolution whereby in case of *P. aeruginosa* the horizontal transfer of mobile genetic elements is the most rapid and extensive process of strain diversification within clonal complexes (see below).

### *Pseudomonas aeruginosa* clone C virulence

The range of infections caused by *P. aeruginosa* clone C strains seems to be as broad as the infection spectrum caused by the entire species. Clone C strains not only colonize the lung in individuals with different underlying etiology such as bronchiectasis (Hilliam *et al*. 2017), but also cause, for example, urinary tract (Tielen *et al*. 2011) and ear infections (Dinesh *et al*. 2003; Curran *et al*. 2004) and are found in the clinical environment (Bosshammer *et al*. 1995). Furthermore, clone C strains are widely distributed as they have been reported to infect CF patients on different continents (Römling *et al*. 1994b; Scott and Pitt 2004; Kidd *et al*. 2011; Fothergill, Walshaw and Winstanley 2012; Middleton *et al*. 2018; Parkins, Somayaji and Waters 2018).

**Figure 1.**
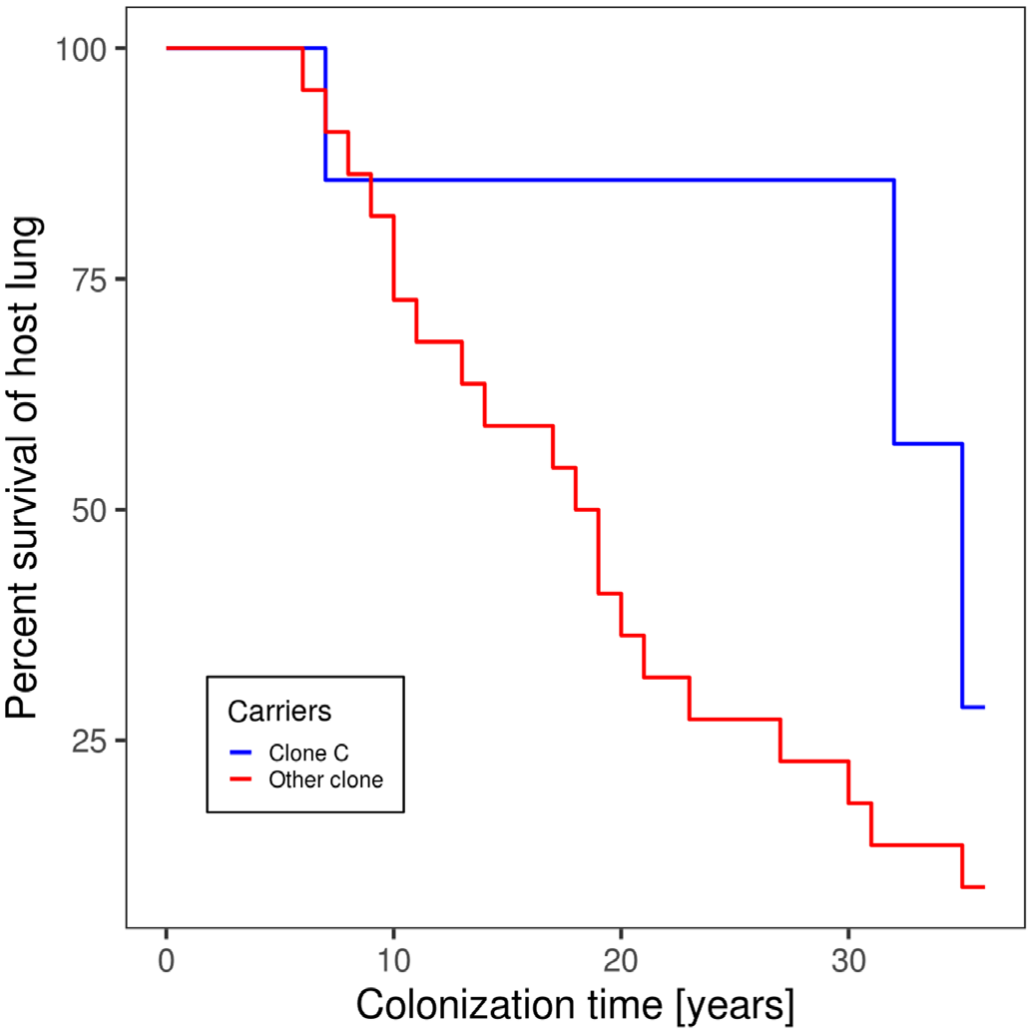
Duration of the chronic airway infection with *P. aeruginosa*in individuals with cystic fibrosis carrying clone C (blue line) or any other clone (red line). The Kaplan–Meier plot shows individual length of colonization until lung transplantation or death because of respiratory insufficiency for the non-transplanted patients until June 1st, 2020. The data was extracted from the medical records of 29 CF patients regularly seen at the CF clinic Hannover who became chronically colonized with *P. aeruginosa* between 1984 and 1990.

Despite its prominent role in the global *P. aeruginosa* population and in acute and chronic infections, clone C has never been mentioned as highly virulent in comparison to other clones such as the PA14 clone D421 (STM253) (Rahme *et al*. 1995; He *et al*. 2004) or the international multidrug-resistant ST235 high-risk clone which has become the most frequent clone of severe acute infections in humans (Treepong *et al*. 2018). Like many other clonal lineages, the clone C genome harbors the genes for virulence factors and pathogenicity traits such as the type III secretion system effector proteins exotoxins S, T and Y or secreted proteases like LasA or LasB, although unconventional regulation of virulence factors by individual isolates has been demonstrated (Kamal *et al*. 2019). The presence of exotoxin S designates clone C as an invasive *P. aeruginosa* clone, in contrast to cytotoxic strains which mutually exclusive bear the patatin-like phospholipase exotoxin U as effector protein. Nevertheless, clone C strains successfully establish chronic, often life-long, infections in the airways of CF patients (Tümmler *et al*. 1991; Römling *et al*. 1994b). However, the chronic clone C carriers, as judged by semi-annually collected P. aeruginosa isolates from respiratory secretions, experience a rather mild course of their *P. aeruginosa* infection without a rapid decrease of lung function. Among the 29 individuals with CF who became chronically colonized with P. aeruginosa during the years 1984–1990 (Cramer *et al*. 2012), six of seven clone C carriers were still alive by June 2020. Conversely, 15 of the 22 carriers of other *P. aeruginosa* clones had passed away indicating that colonization by clone C was associated with a milder outcome of CF lung disease than chronic airway colonization with any other *P. aeruginosa* clone (*P *= 0.018, Fisher's exact test). This difference in the prognosis between clone C and non-clone C carriage is visualized as a Kaplan Meier plot (Fig. 1), which monitors the colonization time of CF airways with *P. aeruginosa* until death or lung transplantation by June 1^st^, 2020.

**Figure 2.**
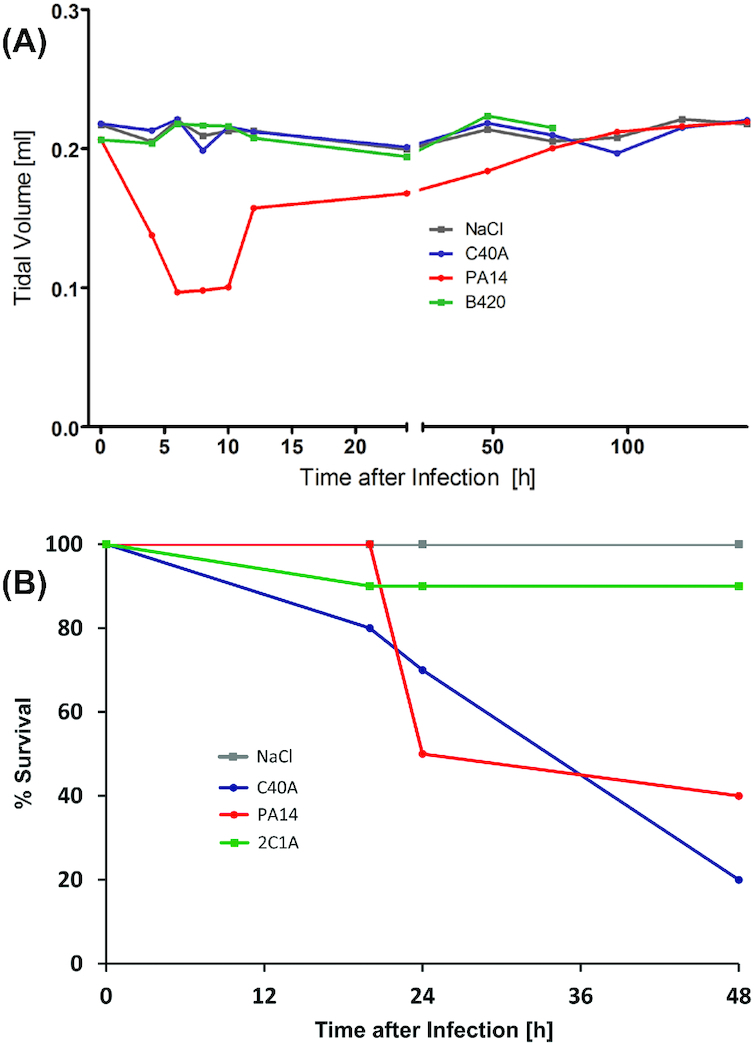
Virulence potential of clone C. The diagrams display the outcome of virulence tests for clone C (here termed C40A) in comparison to other clonal lineages (Hilker *et al*. 2015). The strains were tested in a murine airway infection model and in a wax moth (*Galleria melonella*) larvae infection model. For comparison of the severity of the mouse infection, lung pathology and cytokine responses were monitored and parameters such as body weight or rectal temperature were assessed. In addition, headout spirometry was performed on the infected mice. For negative controls, mice received NaCl solution. As an example for the different degrees of virulence in mouse infection, a diagram of displaying the development of tidal volumes are shown upon infection with clones C/C40A, B420 and PA14 (panel **A**). While clone PA14 inflicted a severe lung infection phenotype, clone C and B420 did not display much virulence potential in this assay. In contrast, clone C displayed high virulence in the *G. melonella* larvae infection model (panel **B**). While for some clones (such as 2C1A) most larvae could overcome the infection, clone C was found among the strains with the highest virulence in this invertebrate assay killing even higher proportions of the infected larvae than clone PA14.

**Figure 3.**
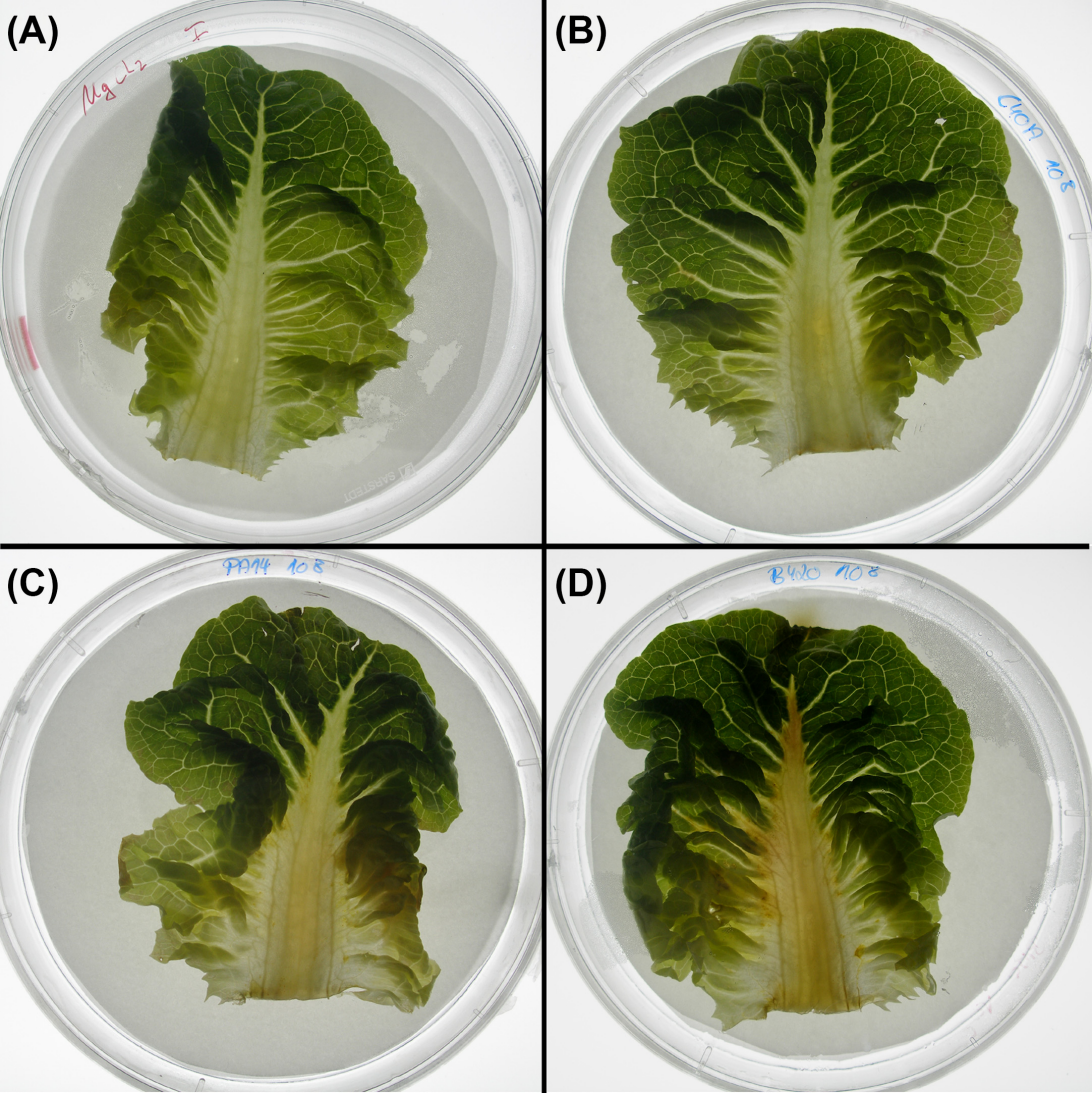
Different degree of virulence in a salad leaf infection model. The examples display the different virulence of *P. aeruginosa* strains in a salad (*Lactuca sativa* var. *longifolia*) infection assay. MgCl_2_ solution containing 10^8^ CFU of bacteria was instilled into the midrib of the lettuce leaf. Progress of infection was represented by the spread of a brownish rotten area to the different parts of the salad leafs. The panels show the spread of the infection 44 h after instillation of MgCl_2_ solution (negative control, panel **A**), clone C/C40A (panel **B**), clone PA14 (panel **C**) and B420 (panel **D**). The respective leaf appeared rather unaffected after instillation of clones C and PA14. Clone B420, however, which displayed very low virulence in the mouse infection model, caused a much more severe phenotype in this assay with rotting visible in wide areas of the lettuce leaf.

Nevertheless, prima vista clone C cannot be described as less virulent. Representative strains of the 15 most common clones and five exclusively environmental clones have been compared in their virulence in three infection models (Hilker *et al*. 2015). In the murine airways with virulence monitored by lung function, ethology and inflammation (Munder and Tümmler 2014) (Fig. 2A), the clone C representative was lowly virulent ranked at positions 15 among 20 tested clones. In the plant infection model of lettuce leafs (*Lactuca sativa* var. *longifolia*) (Starkey and Rahme 2009) (Fig. 3), again the clone C representative was lowly virulent ranked at position 19 among 20 tested clones. Conversely, in the wax moth (*Galleria melonella*) larvae infection model, assessing the proportion of dead larvae (Pustelny *et al*. 2013; Kamal *et al*. 2019) (Fig. 2B), the clone C strain was the third most virulent strain. Likewise, the degree of virulence of clone C representatives in amoeba (Sandström and Römling, unpublished results) was close to that of a PA14 representative. Variable virulence phenotypes were also common for the other clones. These studies exemplarily demonstrated that the pathogenicity of *P. aeruginosa* is context-dependent with clone C demonstrating virulence especially in invertebrate hosts. However, even in one particular model different clonal isolates can show variable virulence properties indicating heterogeneity of the individuals (unpublished results). Nevertheless, the epidemiological evidence is rather strong that human infections with the most common clone C are more benign than those with the high-risk clones.

### Intraclonal genomic sequence diversity

**Figure 4.**
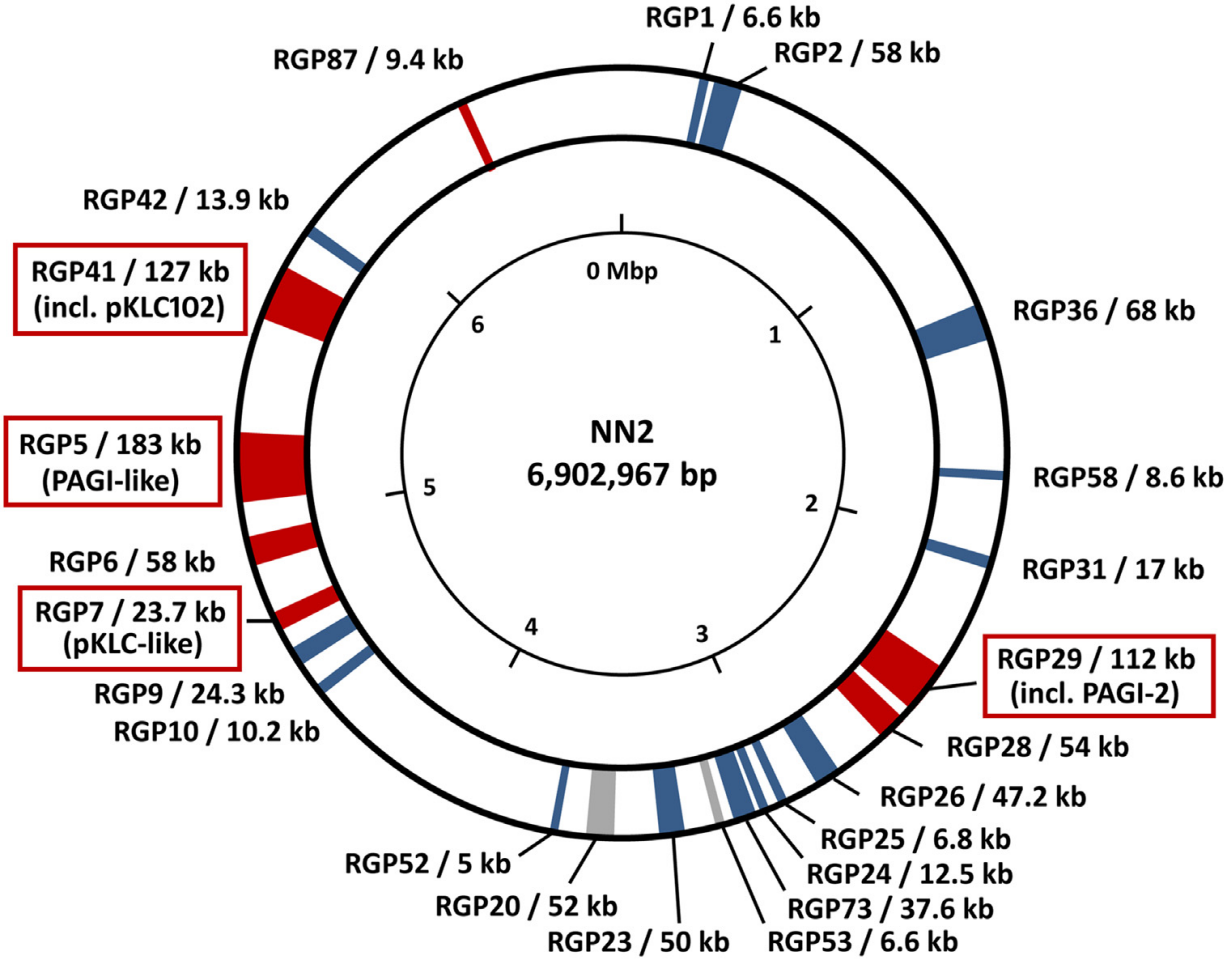
Accessory DNA elements in the genome of the clone C isolate NN2. Regions of the NN2 genome containing accessory DNA are indicated by coloured segments according to conservation in other *P. aeruginosa* genomes. Grey segments indicate accessory DNA occurring in other clone C isolates as well as in other clonal lineages while clone C specific accessory elements are shown in blue. Red segments indicate accessory elements which are fully conserved only in NN2 and closely related isolates but are absent or only partially conserved in other clone C genomes. The accessory elements are tagged by the so-called ‘region of genome plasticity’ (RGP) assignment defining the flanking core genome parts (Mathee *et al*. 2008, Klockgether *et al*. 2011) and the size of the accessory DNA inserted at the respective locus. Tags of RGPs harbouring PAGI-2- or pKLC102-like genomic island are marked by red boxes. Accessory DNA blocks < 5 kbp are not shown in this figure. The 23 displayed accessory elements of isolate NN2 make up for approx. 990 kbp of DNA in total, equivalent to 14% of its chromosomal DNA. The majority of the smaller elements is generally conserved in other clone C strains or even in other clonal lineages while most large accessory elements seem to be specific for the reference or for subsets of clone C isolates. Among them, the largest elements belong to the PAGI-/pKLC-like island family (Klockgether *et al*. 2007) of which three representatives > 100 kbp (RGP29, RGP5, RGP41) and a fourth fragmentary element (RGP7) are present in the NN2 genome.

The median intraclonal sequence diversity among 58 clone C genomes at the single nucleotide level was determined to be 3.7 × 10^−4^ (Fischer *et al*. 2016). Remarkably, the sequence diversity of the core genome was just 8 × 10^−6^ (in comparison: 2 × 10^−5^ for clone PA14 (Fischer *et al*. 2016)), which is more than 100-fold lower than the sequence diversity among unrelated *P. aeruginosa* clones (Hilker *et al*. 2015). In other words, clone C strains differ in their core genome by just a few dozen SNPs from each other and are clearly distinguishable from unrelated clones. The few hot spots of mutations are phage- and plasmid-derived genes and genes encoding the heavy metal ion efflux protein CusA, the cyclic-di-GMP phosphodiesterase BifA and LasR, a key regulator of acyl homoserine lactone quorum sensing. Elements of the accessory genome, i.e. the genomic islands PAGI-2, PAGI-4 and pKLC102 and the regions of genome plasticity (RGPs) 5, 6, 10 and 26 (Fig. 4) (see below for a more extensive description), demonstrated the largest sequence diversity. These elements contain gene clusters, which are characteristic for certain classes of mobile elements. The orthologous conserved elements usually show a nucleotide identity below 98% and therefore typically display a significantly higher number of nucleotide exchanges among the genomes of clonal isolates compared to the core genome backbone genes.

**Figure 5.**
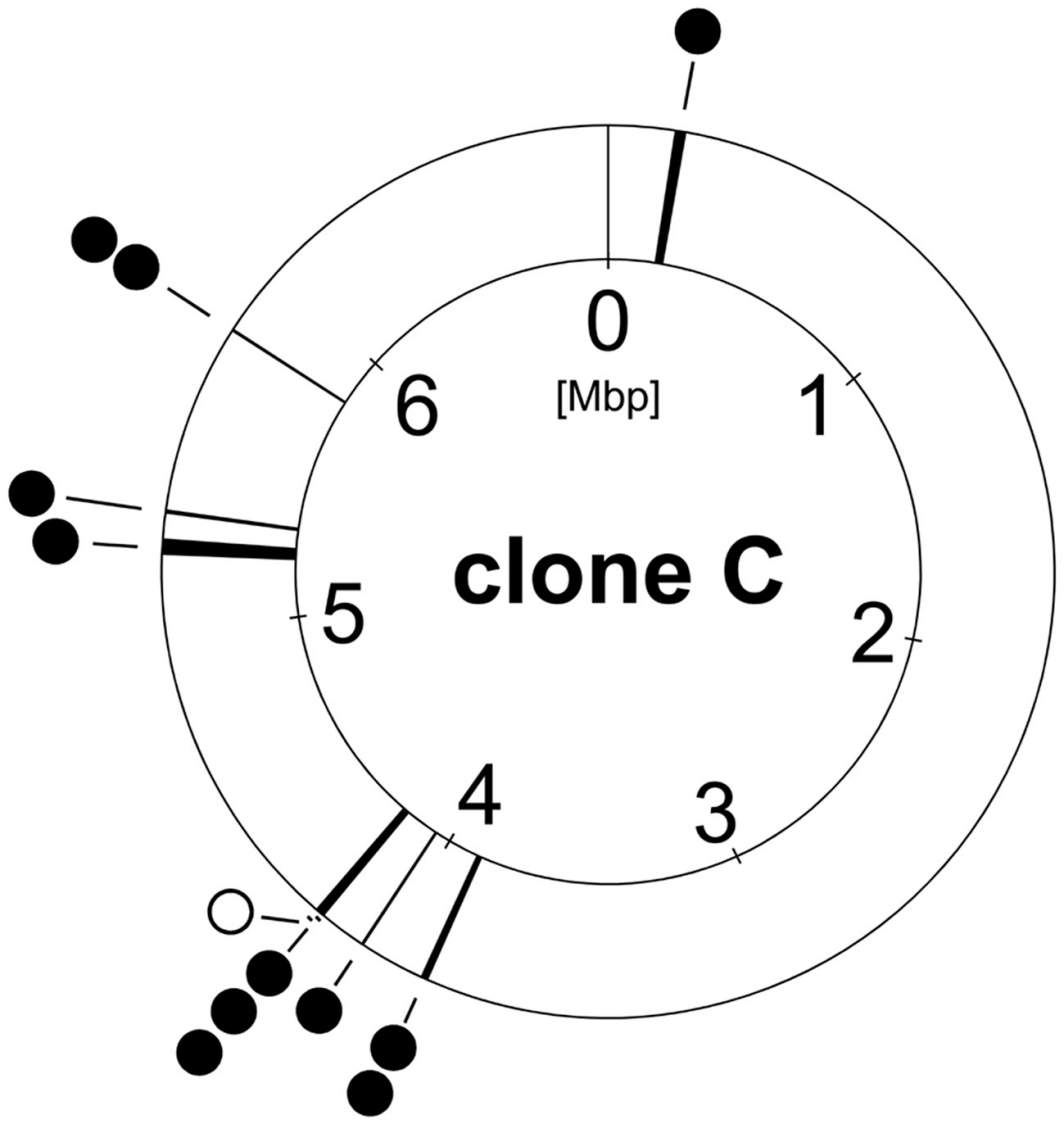
Clone C core genome deletions. Deletions in the core genome found in the clone C strain panel from different habitats (full circle  =  chronic infection, open circle  =  acute infection). Environmental isolates showed no deletions within the core genome.

Consistent with the low intraclonal sequence diversity of the core genome, the length of syntenic segments with 100% sequence identity had a median size of 99 kb between pairs of clone C strains (Fischer *et al*. 2016). Thus the length of 100% pairwise conserved sequences is 1000-fold longer than between unrelated clones (Hilker *et al*. 2015). The chromosomal frame of the core genome is thus conserved among clone C members and only in a few cases disrupted by larger deletions (Fig. 5). However, rapid evolution of the clone C strains’ genome can occur, for example, in the CF lung. Hypermutators, which are impaired in DNA repair or replication fidelity genes and thus possess an up to 1000-fold higher mutation rate, arise in clone C strains during CF lung colonisation (Oliver *et al*. 2000; Kresse *et al*. 2003; Mena *et al*. 2008). As another mechanism of diversification, we observed the expansion of the insertion sequence ISPa20 in the C13 clone C sublineage in one patient (Kresse, Blöcker and Römling 2006). Thirdly, large chromosomal inversions around the origin of replication that conventionally accompany bacterial speciation, creating a CF adapted phenotype have been observed in CF isolates (Römling, Schmidt and Tümmler 1997a; Kresse *et al*. 2003).

**Figure 6.**
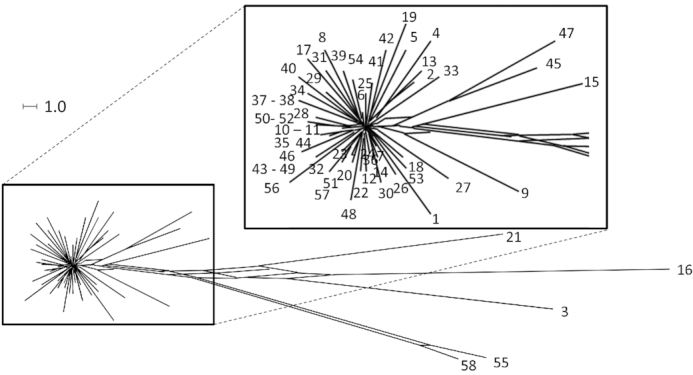
Clone C strain panel dendrogramm. SNP-based phylogenetic trees of the clonal complex C. Only polymorphisms of the core genome were used because mobile elements show a higher variance and are acquired through horizontal gene transfer. The star-like structure visualizes the variability of the core genome by independent de novo mutations. Some outlier strains form a distinct group and will become independent clonal lineages.

Using the syntenic segment length a parameter to assess the relatedness of clone C strains, the majority of strains form a star-like structure of closely related independent singletons of just one strain in a split tree (Fig. 6; (Poigbo, Wolf and Koonin 2012)). A few clone C strains are distant outliers. The preponderance of singletons suggests that most isolates of clone C diverged from a common ancestor by few independent events.

### The NN2 clinical clone C reference genome

The cystic fibrosis (CF) isolate NN2 was selected as the clinical reference strain for clone C. NN2 is the first *P. aeruginosa* clone C isolate from a *P. aeruginosa* naïve subject with CF. The 30-year-long genomic microevolution of the NN lineage was resolved in its CF host until lung transplantation (Cramer *et al*. 2011). The 6902,967 bp large NN2 genome encodes 6601 open reading frames (ORFs), 62 transfer RNAs, 13 ribosomal RNAs and 1 transfer-messenger RNA. Strain NN2 shares 5455 genes with *P. aeruginosa* PAO1. Major phenotype-stratifying differences in coding sequence between NN2 and PAO1 were noted in 39 loci including the adherence sensor *ladS* (Broder, Jaeger and Jenal 2016), genes of pyocyanin and phenazine production (*ptsP*) (Xu *et al*. 2005), protein secretion (*ftsY*) (Ma *et al*. 2003), chemotaxis (*cheR*) (Sheng *et al*. 2019) and biofilm formation (*wspR*) (Huangyutitham, Guvener and Harwood 2013). Strain NN2 harbours a repertoire of 47 inserted elements in its accessory genome. Of the 1246 non-PAO1 ORFs numerous genes may confer specific fitness traits to clone C isolates such as DNA repair genes or heavy metal resistance determinants (see the next sections for a more extensive description of the accessory genome).

5′ untranslated regions (5′-UTRs) are major regulatory components at the mRNA level. Specific secondary RNA structures (aptamers) constitute binding sites for effector molecules, which are part of expression platforms to regulate downstream genes. The 5′UTRs of expressed genes in the NN2 genome were examined by RNA-sequencing (own unpublished data; (D'Arrigo *et al*. 2016)). With 70 nucleotides, the median length of 5′UTRs is similar in NN2 as reported for strain PA14 (Wurtzel *et al*. 2012). However, short 5′-UTRs of 10 to 20 nucleotides in length are more common in the NN2 clone C genome. Those transcripts with short 5’UTRs or even leaderless transcripts lack aptamer-based regulation. When growing under nutrient rich conditions in a fermenter, the 73 NN2 ORFs with the shortest 5’-UTR were expressed at significantly lower mRNA transcript level (*P *< 5 × 10^−5^) compared to the corresponding orthologs with longer 5’-UTRs by the reference strain PA14 (Dotsch *et al*. 2012).

In *P. aeruginosa*, the role of the 5′-UTRs has been investigated for few loci, including genes involved in virulence (*lasB*) (Fukushima *et al*. 1997; Brumlik and Storey 1998), quorum sensing (*rhlA* and *lasI*) (Grosso-Becerra *et al*. 2014), quinolone signaling (*pqsABCDE*) (Brouwer *et al*. 2014) and phenazine synthesis (Li *et al*. 2011). For *lasI*, a 5′-UTR of only 11 bp was detected in the clone C strain NN2 thus lacking the ROSE family RNA thermometer motif described for the 5′-UTR of *lasI* in the reference strain PAO1. As this motif mediates thermoregulation by binding of heat shock proteins (Grosso-Becerra *et al*. 2014), the NN2 should lack this type of temperature regulation for the central quorum sensing autoinducer synthase gene *lasI*.

### The accessory genome of *Pseudomonas aeruginosa* clone C isolates

A *P. aeruginosa* genome typically consists of a single circular chromosome and, in some cases, episomal plasmids. The major part of the circular chromosomes represents the highly conserved ‘core genome’ found in all strains of the species with nucleotide identities > 99%. At various positions, however, DNA blocks specific for subgroups of strains or even single isolates are inserted. The specific DNA blocks typically contain genes derived from phages, plasmids, transposons, insertion elements or other DNA mobility elements such as integrase/transposase genes, DNA helicases, nucleases or genes encoding components of a DNA transfer machinery. Such elements are therefore considered formerly mobile DNA elements acquired by horizontal gene transfer and integrated into the host genome. These DNA blocks described as accessory elements can have an individual size of less than 1000 bp, but can also be as large as > 200 kbp. Genome comparison shows that *P. aeruginosa* genomes typically harbour several dozen accessory elements, which together represent the accessory genome of an isolate. The total size of the accessory genome elements is variable, but usually accounts for more than 10% of the overall genomic DNA of a *P. aeruginosa* strain (Klockgether *et al*. 2011; Freschi *et al*. 2019).

### A special type of accessory elements—PAGI-2/pKLC102 like genomic islands

Initial assessment of the accessory genome of clone C strains by physical mapping revealed a common pKLC102 plasmid in a collection of 21 isolates and the presence of various segments of non-PAO DNA often only present in a subgroup or a single strain (Schmidt, Tümmler and Römling 1996; Römling, Schmidt and Tümmler 1997b). Very large specific DNA elements (> 100 kbp) termed PAGI-2/pKLC102-like islands were predominantly detected at three distinct genomic regions inserted at tRNA genes (Larbig, Kiewitz and Tümmler 2002; Klockgether *et al*. 2004). These genomic islands, which apply a phage like integration mechanism (Kiewitz *et al*. 2000; Burrus *et al*. 2002; Larbig, Kiewitz and Tümmler 2002), also display features of conjugative plasmids. Up to 60 ORFs representing a conserved ‘backbone’ of genes involved in DNA organization and transfer are shared among such islands with nucleotide identity values of 70–100%. The islands also contain blocks of unrelated ‘cargo’ DNA, which confer individual features to the host strains (Klockgether *et al*. 2008). Due to this combination of ‘backbone’ and ‘cargo’ genes, PAGI-2/pKLC102-like islands display a ‘semi-conserved’ composition. Among the conserved backbone genes, many code for yet unknown functions. However, genes with similarity to type IV secretion system components indicate formation of a DNA transfer machinery (Kung, Ozer and Hauser 2010), while other genes code for products involved in the integration and/or excision of the islands such as *parA* or *parB*-like chromosome partitioning or integrase genes.

Annotation of the cargo genes has revealed unconventional physiological traits encoded by the individual islands beyond conventional pathogenicity islands. For example, PAGI-2 of the clinical isolate NN2 harbours genes involved in energy metabolism, such as components of the disulphide bond (dsb) formation system, cytochrome C biogenesis and oxidase proteins; and determinants of heavy metal resistance. PAGI-3 from the environmental strain SG17M has among its cargo putative *pnt* genes encoding nicotinamidenucleotide transhydrogenase proteins and predicted glutamine synthase genes (Larbig, Kiewitz and Tümmler 2002; Lee *et al*. 2014). Cargo genes thus endow the host strain with specific individual metabolic and resistance traits that allows the colonisation of otherwise inaccessible habitats or confer advantages in competition with other strains or species upon colonising a new habitat. In addition, as exemplified with the clone C specific TLPQC island (see below), competitive advantages might not contribute to new traits exclusively encoded in genomic islands. The acquisition of metabolic gene clusters homologous or functionally similar to core genome genes might provide the host strain with extended opportunities such as regulation of carbon and energy metabolism, which provides metabolic fine-tuning and flexibility to adapt to changing environmental conditions. For example, the PAGI-2 genes in the clinical isolate NN2 mentioned above could aid in the protection against oxidative stress as experienced in the CF lung habitat. Similarly, *pnt* genes have been shown to be required for optimal growth and tolerance against ethanol (Kamarainen *et al*. 2017; Long *et al*. 2018; Liu *et al*. 2019) and can potentially be involved in the protection against oxidative stress.

Semi-conserved PAGI-2 or pKLC102-like islands are frequently present in clone C genomes. About 58 genome-sequenced isolates of a clone C collection harbor at least one island, while 32 (55%) of the isolates possess two or even more islands (Fischer *et al*. 2016). Although with lower frequency, PAGI- or pKLC102-like island are also present in *P. aeruginosa* strains from other clonal lineages. Prominent examples are the pathogenicity island PAPI-1 in reference strain PA14 with genes contributing to plant and mouse virulence (He *et al*. 2004) and the ExoU-island A (Kulasekara *et al*. 2006). PAGI-like islands were also detected in other Pseudomonads and in other genera, mainly beta- or gamma-proteobacteria that had been classified in the pre-genomic era as ‘honorary pseudomonads’. These islands might have emerged from an ancestral mobile element, which allowed the uptake/exchange of DNA via horizontal gene transfer between different species and genera. For instance, an identical copy of the PAGI-2 island from a German clinical clone C isolate (Larbig, Kiewitz and Tümmler 2002) was found in a *Cupriavidus metallidurans* isolate from a metal-contaminated environment in Belgium (Mergeay *et al*. 2003). Another example is the *clc* element, which was transferred from *P. knackmussii* B13 to *P. putida* F1 (Ravatn, Zehnder and van der Meer 1998) and *P. aeruginosa* PAO1 (Gaillard *et al*. 2008).

**Figure 7.**
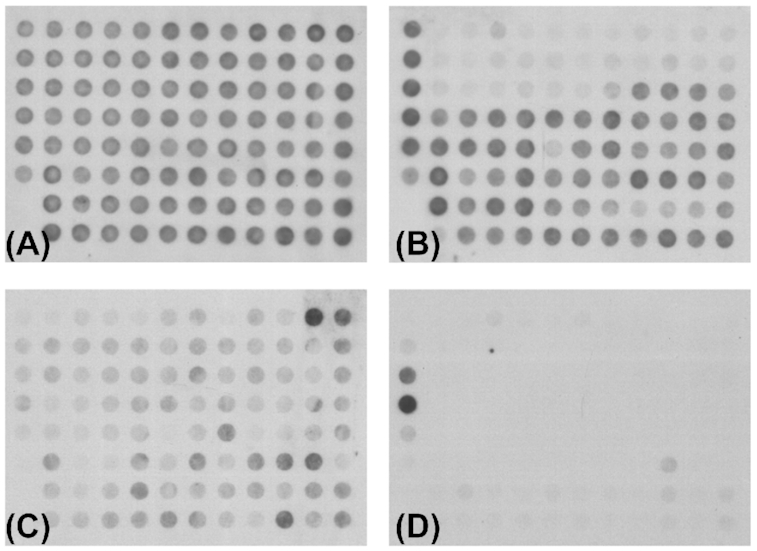
Loss of PAGI-type islands in sequential clone C islands from cystic fibrosis airways. The presence of PAGI-2 or partially related islands in *P. aeruginosa* genomes was tested with hybridisation of macroarrays representing the ORFs located in PAGI-2- Upper row: Hybridisation patterns of clone C strains SG1 **(A)** and SG3 **(B)**, isolated at the onset of a chronic *P. aeruginosa* infection or three months later, respectively. Lower row: Hybridisation patterns for clone C strains NN18 **(C)** and NN86 **(D)**, isolated three or 17 years, respectively, after the onset of a chronic *P. aeruginosa* infection. The patterns for both pairs demonstrate the loss of an island from the host genome in the later isolate while it was still present in the earlier isolate. In case of SG3, the PAGI-2 island itself was lost. Another semi-conserved element is still present but the absence of signals in the upper rows, representing the PAGI-2 specific ORFs, indicate the loss of this island. The weaker hybridisation pattern for NN18 indicates the presence of a related PAGI-like island. This island is apparently absent in the later isolate NN86 as the corresponding hybridisation result displayed only two prominent control spots but no clearly positive PAGI-2 specific signals. This figure and the corresponding results were originally published in Klockgether *et al*., J Bacteriol 2007, Vol 189(6), p. 2443–2459. The reuse of this figure was kindly permitted by the Copyright Holders (Copyright ©2007, American Society for Microbiology).

*Pseudomonas* *aeruginosa* plasmid pKLC102 (Kiewitz *et al*. 2000; Klockgether *et al*. 2008), the *clc* island and their derivatives are integrative and conjugative elements (ICE). The pKLC102 element is present in all clone C strains. Mobilisation from the host chromosome and formation of a circular element with copy numbers up to 30 per host chromosome occurs for pKLC102 in *P. aeruginosa* strain SG17M (Klockgether *et al*. 2007) and consequently made up 10% of the mRNA content (Klockgether *et al*. 2008). Upon mobilisation, the islands were precisely excised from the host chromosome without affecting the surrounding core genome DNA. The retained potential for autonomous replication is consistent with a postulated replication origin (*oriV*) within pKLC102. Also, the *clc* island can excise from the chromosome and form a circular intermediate in which both ends are connected (Sentchilo, Zehnder and van der Meer 2003). Circular isoforms could not be detected in clone C strains for PAGI-2 and PAGI-3 islands under laboratory conditions (Klockgether *et al*. 2007), but chromosomal excision can occur at low frequency in sequential isolates. Loss of PAGI-2 from the chromosome was observed in serial clone C isolates that had been retrieved from the airways of a CF patient in half-year intervals (Fig. 7). A comparably precise excision as seen for the excision/mobilisation of pKLC102 can be postulated.

Individual clone C genomes may harbor several PAGI-like islands (Klockgether *et al*. 2007; Wiehlmann *et al*. 2007). For example, the sequenced clinical isolate NN2 carries pKLC102, PAGI-2 and a hybrid of two PAGI-like elements (Fischer *et al*. 2016) and the aquatic isolate SG17M pKLC102, PAGI-3 and PACGI-1 (Lee *et al*. 2015). Interestingly, pKLC102 and PACGI-1, or variants thereof, were detected in all clone C genomes analysed so far. In NN2, the ‘hybrid’ island is highly similar to the SG17M PACGI-1 with approx. 73 kb of conserved DNA containing not only the typical ‘backbone genes’, but also ‘cargo’ genes such as TLPQC-1 genes assigned to protein homeostasis (Lee *et al*. 2015). These closely related islands were both inserted at tRNA_Gly_ genes, but are located in different genomic areas: in SG17M PACGI-1 is found at RGP27, while in NN2 the ‘hybrid’ island with the counterpart is found at RGP5. So, apparently both the clinical and the environmental isolate, or a common precursor strain, took up a similar accessory element. The element can be either integrated at different sites into the chromosome a priori, or after horizontal transfer into an ancestor clone C stain changed its location by subsequent transposition events. If chromosomally integrated, pKLC102 is alternately inserted at RGP41 or RGP7 into a tRNA_Lys_ gene.

After integration into the host genome PAGI-like islands can rapidly diversify by nucleotide substitutions, insertions or deletions. Secondary insertions of IS elements or transposons generate a mosaic-like architecture of the islands. For example, a transposon and remnants of pKLC102 were assembled to genome island PAGI-4 in strain NN2. Due to these secondary events pKLC102 sequences may become irreversibly fixed in the chromosome (Romling *et al*. 1997) as has been seen in a subgroup of clone C strains from CF airways which integrate a hybrid of class I integron, IS elements and aminoglycoside resistance gene cassette called TNCP23 into their pKLC102 sequence (Klockgether *et al*. 2004).

These secondary insertions into PAGI-like islands triggered large chromosomal inversions in some clinical CF clone C strains (Römling, Schmidt and Tümmler 1997a). The inversion breakpoints were mapped to an IS element at the border of the TNCP23 element in the pKLC102 sequence (Kresse *et al*. 2003). Copies of the IS element were identified at both recombination breakpoints indicating that the duplication of the IS element and its subsequent integration at a CF-relevant genomic locus might have initiated the inversion of several Mbp of DNA. There does not seem to be a specificity in the IS elements that can provide the basis for large chromosomal inversions (Kresse, Blöcker and Römling 2006). However, the duplication of an IS element is not mandatory to generate an inversion. In another strain, we have localized the inversion breakpoints in conserved DNA blocks of two PAGI-2 like islands, but no IS element or equivalent cover the breakpoint loci (unpublished data).

### Accessory elements of *Pseudomonas aeruginosa* clone C clinical reference strain NN2

Elements of the accessory genome other than the PAGI-like islands are typically smaller in size. Many of them contain only few ORFs. For example, of the 47 accessory elements in the NN2 clone C genome that distinguish it from *P. aeruginosa* PAO, 24 elements are smaller than 5 kbp (Table 2). Forty-five of the 47 blocks were found in one of the 89 so-called ‘regions of genome plasticity’ (RGPs), loci already defined as candidates for harbouring accessory elements upon genome comparisons of *P. aeruginosa* strains (Mathee *et al*. 2008; Klockgether *et al*. 2011). Whereas the larger PAGI-like islands are specific for a strain or subgroup of clone C strains, the majority of the other elements is shared among all tested clone C strains and likely constitutes the clone-specific signature of the accessory genome. Of the latter group, the element inserted at RGP24 harbours genes that are annotated as CRISPR-related *cas* and *csy* genes that are part of a CRISPR/Cas system in clone C strains similar to the one described for the *P. aeruginosa* reference strain PA14.

**Table 2.** Accessory elements detected in the genome sequence of clone C strain NN2.

Region**^1^**	Size [kbp]	No. of ORFs	Comment
RGP46	1.6	3	
RGP1	6.6	6	
RGP2	58	39	with type I restriction modification system genes
RGP66	3.4	4	phage resistance and type I restriction modification genes
RGP3	2.8	4	
RGP4	4.1	6	
RGP5	183	175	combination of two PAGI-2-like integrated elements^2^
RGP6	58	64	with *trb* conjugative transfer gene cluster
RGP7	23.7	24	PAGI-4, composed of pKLC102 fragment and transposon DNA
RGP9	24.3	22	flagella glycosylation genes^3^
RGP47	2.5	2	with gene encoding for S-type pyocin
RGP10	10.2	16	
RGP11	1.8	2	
RGP48	2.8	4	
RGP13	2.3	2	
RGP15	2.7	3	
RGP76	1.6	2	
RGP52	5	4	
RGP20	52	51	conserved in many clonal linages, also present in strain PAO1
RGP22	2.9	5	
RGP23	50	44	PAGI-1^4^, present in many clonal lineages but absent in PAO1
RGP53	6.6	4	
RGP73	37.6	10	pyoverdine biosynthesis genes^3^
RGP24	12.5	8	with CRISPR-related *cas* and *csy* genes
PA2425/28	2.1	2	pyoverdine biosynthesis genes *pvdS* and *pvdY*
RGP25	6.8	4	
RGP26	47.2	54	with *trb* conjugative transfer gene cluster
RGP71	1.6	1	
RGP28	54	44	with phage like genes
RGP43	2.2	4	
RGP56	3.2	3	
RGP29	112	126	PAGI-2 plus additional 7 kbp accessory element
RGP31	17	17	LPS biosynthesis genes (O-antigen, defining serotype)^3^
RGP58	8.6	6	
PA3576/78	2.3	3	
RGP36	68	59	with *trb* conjugative transfer gene cluster
RGP89	0.6	1	
RGP68	2.4	3	with *exoS* and ExoS chaperone gene
PA4092/93	3.2	3	
RGP44	2		
RGP39	4.2	5	
RGP60	0.7	1	major pilin gene *pilA*^3^
RGP41	127	131	integrated element pKLC102, with add. 23 kbp integron
RGP42	13.9	18	with phage like genes
PA5085/90	4.9	4	
RGP87	9.3	10	
RGP80	4.2	5	

1 Accessory elements with a size ≥ 0.5 kbp and at least one annotated ORF are listed.

If the accessory DNA was located in an already defined region of genome plasticity, the respective RGP no. is given. Other loci are described by the flanking core genome genes (designations of homologs from reference strain PAO1 are given).

2 One of the two elements is highly similar to the element PACGI-1 from the environmental strain SG17M, which is located in a different region of the chromosome there (RGP27).

3 So-called replacement island (see Table 2)

4 First described *P. aeruginosa* genomic island (Liang *et al*. 2001); element not related to other PAGI-2-like elements

### *Pseudomonas aeruginosa* clone C replacement islands

A specific subgroup among the accessory genome elements are four clusters of functionally well described genes that are present in all *P. aeruginosa* strains. Genes for LPS biosynthesis (defining the serotype), pyoverdine biosynthesis, flagella glycosylation and the major pilin PilA are found in all genomes, but, unlike core genome parts, are variable elements within the species. The respective counterparts in different strains cannot only be discriminated by high nucleotide substitution rates, the clusters can also differ in gene composition and size. In contrast to accessory elements each genome carries one version of each of these four gene clusters, which are, independently of the respective type or subtype, always located at the same position within the conserved core genome. These gene clusters have been termed replacement islands, which have developed under diversifying selection early in the evolution of *P. aeruginosa* (Kung, Ozer and Hauser 2010). Within clonal lineages, the subtypes of the four replacement islands are conserved. As listed in Table 3, the clone C reference strain NN2 harbours a LPS biosynthesis serotype 01 gene cluster (Raymond *et al*. 2002) and a type a1 flagella glycosylation cluster (or ‘a-type long’) (Arora *e al*. 2004). The pyoverdine biosynthesis gene cluster can be assigned to type II (Smith *et al*. 2005). The major pilin gene *pilA* of clone C strains belongs to group II (Spangenberg *et al*. 1995; Kus *et al*. 2004).

**Table 3.** Replacement island types of *P. aeruginosa* clone C^1^.

Gene (cluster)	RGP**^2^**	Detected type	Reference	Comment
LPS biosynthesis (O-antigen)	RGP31	serotype 01	(Raymond *et al*. 2002)	
flagella glycosylation	RGP9	a-type long (a1)	(Arora *et al*. 2004, Schirm *et al*. 2004)	
pyoverdine biosynthesis	RGP73	type II	(Smith *et al*. 2005)	comparably high nucleotide identities (99.1%–99.5%) with all three subtypes (IIa, IIb, IIc) defined in the reference paper
major pilin (*pilA*)	RGP60	type II	(Voisin *et al*. 2007)	assignment to type II due to shared gene synteny in this region
exotoxin S	RGP68	*exoS*	(Kulasekara *et al*. 2006)	so far no strain was found sharing all clone C markers but harbouring an exoU gene at RGP7^3^

1 Determination of types based on predicted genes from the genome sequence of clone C strain NN2. Types are usually conserved within a clonal lineage.

2 Genomic location according to definition of Regions of Genome Plasticity (RGPs) (Mathee *et al*. 2008, Klockgether *et al*. 2011).

3 In *P. aeruginosa* genomes exotoxin S or exotoxin U genes do not occur at the same location but in individual RGPs (*exoS*: RGP68, *exoU*: RGP7); similar to the other replacement islands, however, presence of an exotoxin S or U gene cluster is mutually exclusive.

### Variation of the gene repertoire within *Pseudomonas aeruginosa* clone C

The strain-specific acquisition of genes generates traits that modulate fitness, virulence, lifestyle or metabolic competence on the level of the individual isolate. An average clone C strain harbors about 100 strain-specific genes in its accessory genome (Fischer *et al*. 2016) (Table S1, Supporting Information). This gene pool is primarily acquired from phylogenetically related bacteria. For about 80% of these genes the closest orthologues were identified in other *P. aeruginosa* clones or other *Pseudomonas* species (Table 4). According to database searches, a further 20% of the genes have their closest homologue among other gamma- (8.7%) or beta-proteobacteria (11.5%) such as *Haemophilus somnus*, *Klebsiella pneumoniae, Salmonella enterica, Achromobacter piechaudii, Achromobacter xylosoxidans* or various *Burkholderia* species (Fischer *et al*. 2016).

**Table 4.** Numbers of strain-specific genes detected in a panel of 58 clone C strains.

		No.	% of Total No.	Median No. per Strain
In 58 strain panel	7488	100	104	
Closest Homolog in				
	other P. aeruginosa	4349	58.08	71
	other Pseudomonads	1620	21.63	14
	other γ-proteobacteria	654	8.73	4
	other origin	865	11.55	11

In contrast to other common *P. aeruginosa* clones, clone C strains have enlarged their genetic repertoire for carbohydrate metabolism. *P. aeruginosa* typically prefers amino acids and fatty acids as carbon source, but this repression of the uptake and catabolism of sugar (‘catabolite repression control’) (Linares *et al*. 2010) does not apply to the most common clone C strains that may compensate the core genome-predetermined limitations in the utilization of sugars by the horizontal acquisition of genes of carbohydrate metabolism (unpublished results).

### Phenotypic variability in *Pseudomonas aeruginosa* clone C strains

Phenotypes, the timely expression of genetic information, and the regulation of phenotypic traits by environmental and internal signals have been studied mainly in the model strains *P. aeruginosa* PAO and PA14, both clinical isolates. Thereby, sophisticated regulatory mechanisms, for example, for the secretion of effector proteins of the type III secretion system upon removal of the divalent cation Ca^2+^, have been unravelled (Lee *et al*. 2010). However, the generality of regulatory patterns of phenotypic traits within *P. aeruginosa* has not been established. We exemplarily investigated the expression of two phenotypic traits of *P. aeruginosa*. Although almost equally virulent as PA14 in the wax moth *G. melonella* model system, the aquatic isolate SG17M does not secrete type III secretion system effector proteins under promiscuous conditions (Kamal *et al*. 2019). Variable secretion of type III effector proteins was observed among clinical isolates of clone C and strains of other clonal lineages, however, environmental isolates of clone C, in contrast to non-clone C isolates, did consistently not secrete type III effectors under promiscuous conditions. Likewise, the secretion of the siderophore pyoverdine was not pronounced in environmental isolates of clone C, while two of three clinical isolates showed distinct secretion. However, the ability to produce and secrete pyoverdine is not impaired as pyoverdine secretion is relieved upon deletion of the membrane-bound protease FtsH in the environmental isolate SG17M (Kamal *et al*. 2019). The underlying molecular regulatory mechanisms of phenotypic variability are to be unravelled, with the reduced variability in clonal isolates to provide a more stringent genetic background, which will facilitate the genetic characterisation.

### Microevolution of *Pseudomonas aeruginosa* clone C during chronic infection of CF airways

The colonization of CF airways with *P. aeruginosa* is one of the few opportunities to observe the microevolution of a bacterium during chronic infection in real life (Marvig *et al*. 2015; Winstanley, O'Brien and Brockhurst 2016). When *P. aeruginosa* conquers the CF lungs, the aquatic bacterium needs to adapt phenotype and genotype to a hostile environment characterized by a plethora of nutrients, but also a large battery of deadly host defenses (Tümmler and Kiewitz 1999; Folkesson *et al*. 2012; Moradali, Ghods and Rehm 2017). The microevolution of *P. aeruginosa* clone C strains in CF lungs has been investigated by phenotyping and whole genome sequencing of serial isolates from two patients collected from the onset of chronic colonization over a period of up to 30 years (Cramer *et al*. 2011; Klockgether *et al*. 2018). Both patients who became chronically colonized with *P. aeruginosa* clone C during childhood in the early 1980s had developed rather mild clinical phenotypes. Genome sequencing uncovered from a few hundred to close to a thousand de novo mutations in the serial isolates from the two patients. In parallel, the accessory genome was modified by the loss and acquisition of several DNA blocks. During colonization of the CF lungs isolates became deficient in the secretion of virulence effectors and siderophores. Hence the genotypic and phenotypic conversion of clone C strains is similar to that seen with other *P. aeruginosa* clones (Marvig *et al*. 2015; Klockgether *et al*. 2018).

### A transmissible locus for protein quality control in *Pseudomonas aeruginosa* clone C strains

**Figure 8.**
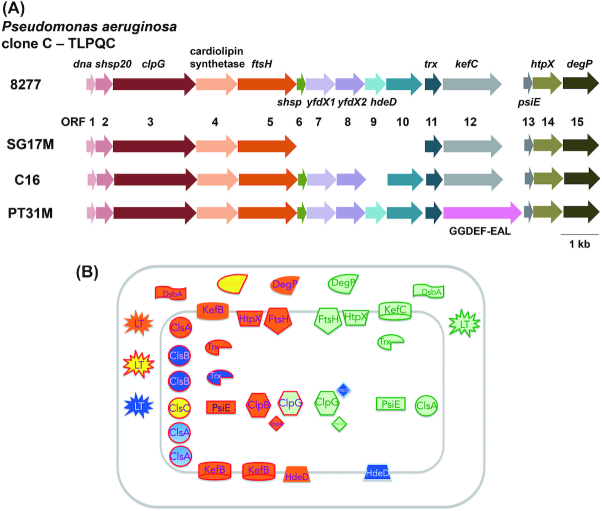
Context of the TLPQC locus in *P. aeruginosa* clone C strains. **(A)** Genetic maps of TLPQC loci in selected clone C strains. TLPQC genetic maps from 8277, a clinical isolate from human urine; SG17M, an environmental clone C isolate from river water in Germany; C16, an isolate from a cystic fibrosis patient (unpublished) and PT31M, an isolate from drinking water (unpublished) are described. Open reading frames 1–15 code for the following proteins: Dna, MerR-like transcriptional regulator; sHsp20, small heat shock protein; ClpG, disaggregating chaperone; Cls, cardiolipin synthase; FtsH, metalloprotease; sHsp, small heat shock protein, YfdX1 and YfdX2, antibiotic resistance, anti-virulence protein; HdeD, transmembrane protein involved in acid tolerance; ORF10, hypothetical protein; Ttrx, thioredoxin; KefC, glutathionine-dependent potassium-efflux system and methylglyoxal detoxification; PsiE, putative phosphate starvation-inducible protein; HtpX, inner-membrane associated peptidase; DegP, periplasmic protein with protease and chaperone activity. In PT31M, *kefC* is replaced by a GGDEF-EAL domain protein encoding ORF. **(B)** TLPQC locus proteins and homologous core genome gene products. Red line frame, core genome gene products; green line frame, TLPQC gene products of SG17M; blue line frame, TLPQC gene products additionally present in other clone C strains. The *P. aeruginosa* clone C core genome genes code for one ClpG disaggregase (and the functional homologue ClpB), one FtsH protease, one HtpX protease, one PsiE protein, two DegP-like proteases (AlgW and MucD) and two thioredoxins (Trx1 and Trx2). Furthermore, genes for three KefB-like transporters and six cardiolipin synthase (CLS) and CLS-like proteins are present on the core genome. All these gene products have counterparts on the SG17M TLPQC locus. TLPQC gene products of other *P. aeruginosa* strains such as the acid resistant protein HdeD, an integral membrane protein, have also counterparts on the SG17M core genome. In addition, genes for a thiol disulfide oxidoreductase DsbA and lytic transglycosylase (LT), also with homologues on the core genome, are encoded elsewhere on PACGI-1.

Genomic islands determine strain-specific traits which are beneficial in terms of virulence, antibiotic resistance, symbiosis, metabolic diversity and adaptation (Juhas *et al*. 2009). In the aquatic strain SG17M, the *P. aeruginosa* clone C-specific genomic Island 1 (PACGI-1, a hybrid of two PAGI-like elements; see accessory genome chapter above) is 86 kb in length encoding over 100 genes (Lee *et al*. 2015, 2016). One border of PACGI-1 constitutes a cluster of protein quality control genes, coding for various holding and disaggregating chaperones, heat-inducible proteases such as FtsH, DegP and HtpX, thioredoxin and other stress resistance genes that seems to be present in all clone C strains (Fig. 8) (Lee *et al*. 2016). Flanked by mobile elements, this gene cluster named as ‘Transmissible Locus for Protein Quality Control’ (TLPQC-1) (Lee *et al*. 2016) and alternatively ‘locus of heat resistance’ (LHR) is consistent with protein homeostasis as a major determinant of temperature tolerance (Mercer *et al*. 2015; Boll *et al*. 2017).

Protein homeostasis is essential for all living organisms (Hartl, Bracher and Hayer-Hartl 2011; Valastyan and Lindquist 2014) and partially determines cell aging and longevity (Koga, Kaushik and Cuervo 2011). Impairment of protein homeostasis by massive misfolding and aggregating processes results in various proteotoxic human diseases such as Parkinson's, Alzheimer's and Huntington's disease and is connected to cell aging and cytotoxicity (Ross and Poirier 2004). In bacteria, survival of various stresses, antibiotic resistance, adaptation, but also physiological processes such as biofilm formation and virulence, are closely associated with protein homeostasis mechanisms (Marr *et al*. 2007, Neckers and Tatu 2008, Lee *et al*. 2016, Pu *et al*. 2019).

**Figure 9.**
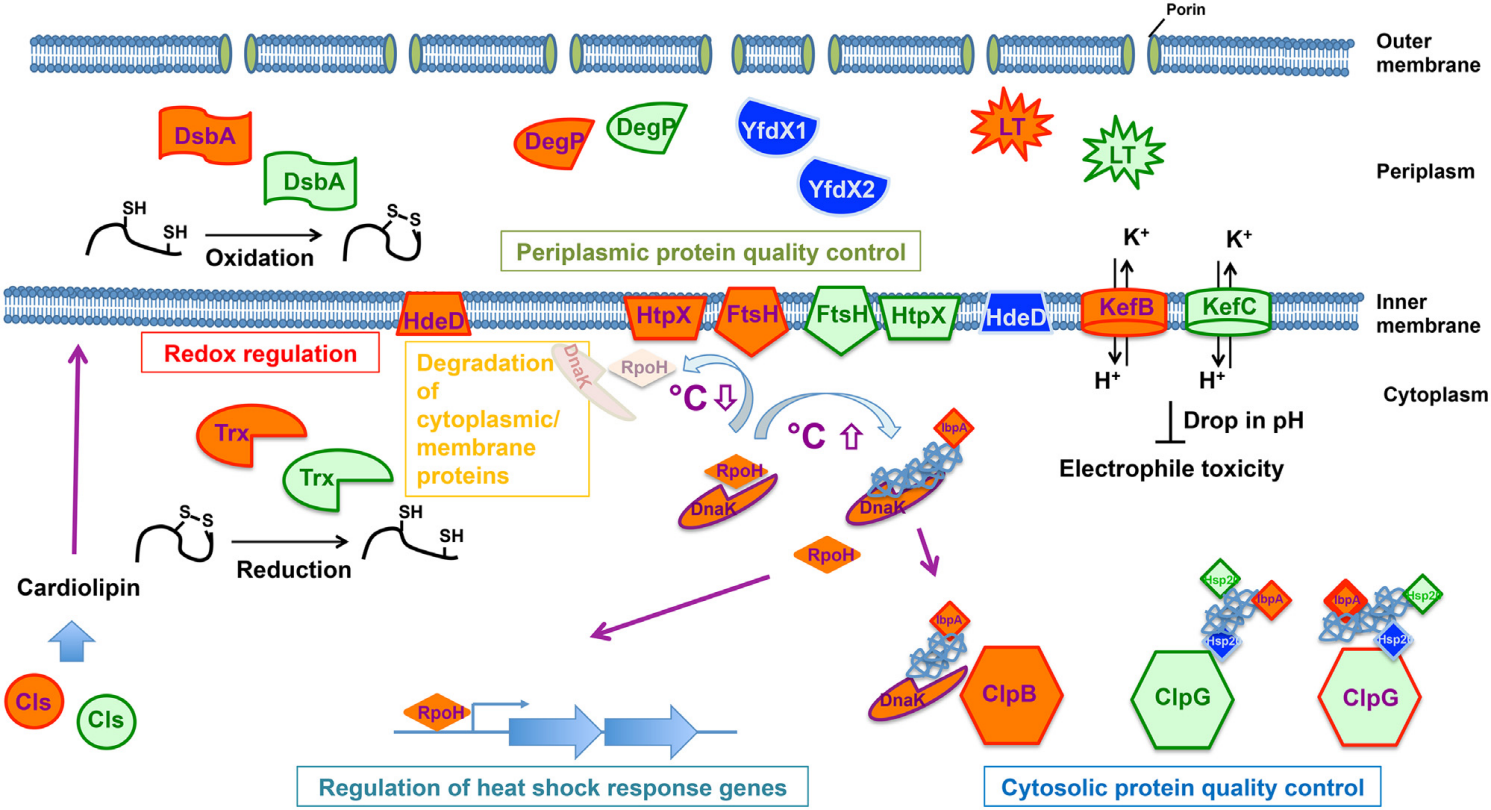
Core functionalities of TLPQC and core genome gene products. Regulation of cytosolic and periplasmic protein quality control, degradation of cytosolic and membrane proteins and redox regulation are major pathways supported by TLPQC gene products. Regulation of the heat shock response by degradation of RpoH is mainly conducted by the core genome membrane-bound protease FtsH. Gene products are described in Fig. 8. For simplicity, one homologous core gene product is displayed.

Recent studies have shown that a potent protein quality control system present on an unconventional genomic island contributes to successful survival and adaptation of *P. aeruginosa* clone C strains with most molecular mechanisms still to be explored in detail (Figs 8 and 9). In this section, we describe the general characteristics of bacterial protein quality control systems as well as report on the initial characterisation of selected gene products of the horizontally acquired clone C specific components for protein quality control.

### The chaperone system for protein quality control in bacteria

Environmental stresses such as elevated temperature, detergents, organic solvents and oxidative stress, unfold and denature proteins and eventually lead to reversible and irreversible protein aggregation. Protein (and small molecule) chaperones are key players in protein quality control systems involved not only in folding proteins, but also in preventing protein aggregation and disaggregating protein aggregates for refolding or degradation (Kim *et al*. 2013; Balchin, Hayer-Hartl and Hartl 2016; Schramm, Schroeder and Jonas 2019). Hydrophobic and electrostatic interactions direct the interactions between chaperones and their client proteins (Kim *et al*. 2013; Koldewey *et al*. 2016; Lee, Kim and Bardwell 2018). The central role of chaperones is shown as, for example, Hsp70 (DnaK), Hsp90 (HtpG) and small heat shock proteins are well conserved among all living organisms (Balchin, Hayer-Hartl and Hartl 2016; Mogk, Ruger-Herreros and Bukau 2019).

In the first instance, chaperones are involved in the *de novo* folding of proteins in the cytosol (Balchin, Hayer-Hartl and Hartl 2016). In bacteria, the nascent peptide chains released from the ribosome are firstly engaged by ribosome-associated trigger factor and the DnaKJE system. Trigger factor is an ATP-independent chaperone. The DnaKJE system consists of Hsp70 (DnaK), its co-chaperone Hsp40 (DnaJ) and nucleotide exchange factor GrpE. The *de novo* folding of protein is subsequently accelerated by the folding chaperones GroESL and HtpG in a process requiring ATP hydrolysis (Balchin, Hayer-Hartl and Hartl 2016).

Upon stress conditions that eventually lead to protein aggregation, small heat shock proteins (sHsps) act as holding chaperones by forming a complex with client proteins to prevent irreversible protein aggregation (Mogk, Ruger-Herreros and Bukau 2019), but also facilitate the resolublization of aggregated proteins through disaggregating chaperones (Mogk *et al*. 2003; Mogk, Ruger-Herreros and Bukau 2019). Under stress conditions such as elevated temperature, changes in the secondary and tetrary structure of sHsps lead to a higher binding affinity to client proteins (Mogk, Ruger-Herreros and Bukau 2019).

Once protein aggregates are formed, the ClpB/Hsp100 disaggregating chaperone system unfolds protein aggregates in an ATP-dependent manner cooperatively with sHsps and the DnaKJE system (Mogk, Kummer and Bukau 2015; Mogk, Bukau and Kampinga 2018). ClpB possesses two asymmetric ATPases associated with diverse cellular activities (AAA+) domains and a coiled-coil structured middle domain (M-domain). The M-domain interacts with aggregate-loaded DnaK to elevate the ATPase and disaggregating activity of ClpB (Mogk, Kummer and Bukau 2015). The ATP hydrolysis generates the threading power to unfold the aggregates and pass the peptide chain into the ClpB hexamer (Mogk, Kummer and Bukau 2015).

Although ATP is lacking in the bacterial periplasmic space in contrast to the cytoplasm, diverse ATP-independent chaperones have been identified (Stull, Betton and Bardwell 2018). As porins in the outer membrane allow free diffusion of small molecules less than 600 Da from the extracellular space (Nikaido 2003), periplasmic proteins are more directly exposed to environmental stress than cytoplasmic proteins. While SurA, Skp and DegP can fold outer membrane proteins (McMorran, Brockwell and Radford 2014), Spy and HdeAB are stress-responsive chaperones responding to alcohol and acid stress, respectively (Stull, Betton and Bardwell 2018).

*Escherichia coli* has been a model organism to investigate the biochemical and physiological role of chaperones. Although *P. aeruginosa* is a key human pathogen with protein homeostasis as a potential target for antimicrobial treatment, the chaperone system has rarely been adressed in this species. With 5.5–7 Mbp, the genome size of *P. aeruginosa* is larger than the 4.5–5.5 Mbp of *E. coli* (Schmidt, Tümmler and Römling 1996; Lee *et al*. 2006; Lukjancenko, Wassenaar and Ussery 2010; Gordienko, Kazanov and Gelfand 2013). Especially, the successful clonal groups of *P. aeruginosa* including PA14 and clone C have distinct genome characteristics such as to display instant double crossover homologous recombination and to flexibly acquire genomic islands through horizontal gene transfer (Römling, Schmidt and Tümmler1997b; Fischer *et al*. 2016; Lee, Kamal and Römling 2019). These observations suggests *P. aeruginosa*, especially common clonal strains, to possess a large cargo of distinct genes, which may include unique genes involved in protein homeostasis, allowing them to survive successfully under a variety of host and environmental conditions. Of note, those gene products, such as disaggregases, might serve as potent disaggregases also for aggregates causing the above mentioned human diseases such as Alzheimer (Gao *et al*. 2015).

### The TLPQC-1 island of *Pseudomonas aeruginosa* clone C encodes xenologues of core genome genes

As a remarcable hallmark of TLPQC, many of the encoded gene products are xenologues of conserved core genome genes of *P. aeruginosa* (discussed below; Fig. 8B; (Lee *et al*. 2015, 2018)). TLPQCs islands, classified in three classes with minimal diversification, but variable gene content, are found not only in common clones of *P. aerguinosa* such as clone C and clone J, but also in clinical and food-derived strains of species from diverse genera, such as *E. coli*, *K. pneumoniae* and *Cronobacter sakazakii* (Bojer *et al*. 2010; Lee *et al*. 2016; Nguyen *et al*. 2017). However, the origin of TLPQC is most likely an environmental bacterium such as *Cupriavidus necator* (*Ralstonia eutropha*) thriving under extreme conditions. TLPQC encoded on either the genome or a plasmid can be efficiently transferred by horizontal gene transfer (Lee *et al*. 2016; Nguyen *et al*. 2017) and integrated into the proximal region of a tRNA gene, a common insertion site of a genomic island. Consistently, in clone C strains, PACGI-1/TLPQC is inserted at the 3′ end of a tRNA_Gly_ gene. However, in SG17M, a strain isolated from river water in Germany, TLPQC is uniquely inserted into the 7th spacer region of the CRISPR locus that encodes an adaptive bacterial immune system against plasmid and phages (Lee *et al*. 2015).

Despite of efficient horizontal gene transfer, only 2% of sequenced *E. coli* strains contain TLPQC, suggesting a detrimental effect in strain backgrounds beyond phylogroup A (Mercer *et al*. 2015). Of note, after preheating of milk to 55–60°C, 36% *E. coli* contained TLPQC (Boll *et al*. 2017). Equally, only 5% of the *E. coli* strains have been found TLPQC positive in untreated wastewater, but 59% *E. coli* strains were TLPQC positive in chlorine-treated wastewater (Zhi *et al*. 2016). These results strongly suggest that TLPQC is selected to aid survival upon protein stress. Consistently, *P. aeruginosa* clone C strains exhibit higher tolerance against lethal heat stress than other epidemic and non-epidemic *P. aeruginosa* strains which do not harbour TLPQC (Lee *et al*. 2015). The core unit of TLPQC is composed of the three genes *dna, shsp20_GI_* and *clpG_GI_* (Lee *et al*. 2016; Nguyen *et al*. 2017). Overexpression of the *dna-shsp20_GI_*-*clpG_GI_* operon can potently enhance heat tolerance even in unrelated *P. aeruginosa* strains, suggesting that these genes are crucial elements for heat tolerance (Lee *et al*. 2015).

### The small heat shock protein sHsp20_GI_

The sHsp20_GI_ belongs to class B of small heat shock proteins in conjunction with other bacterial and fungal small heat shock proteins. Within the B subclass, sHsp20_GI_ is the founding member of a subfamily of TLPQC encoded sHsp20s, horizontally transferred small heat shock proteins of bacterial isolates of various species. Structural modeling showed that a core of two anti-parallel β-sheets consisting of seven anti-parallel β-strands characteristic for the α-crystallin core region of sHsp family proteins is also present in sHsp20_GI_. sHsp20_GI_ uniquely has an extended N-terminal region compared to the well-characterized class A sHsps EcIbpA, EcIbpB and PaIbpA (Lee *et al*. 2015). Biochemical analyses and electron microscopy indicated that the sphere-like sHsp20_GI_ oligomer, probably constituting an inactive state, is composed of 24 monomers consistent with the amino acid sequence core regions II and I required for dimerization and oligomerization, respectively (Lee *et al*. 2015). sHsps typically alter their oligomeric state upon stress such as elevated temperature (Mogk, Ruger-Herreros and Bukau 2019). Although the secondary structure of sHsp20_GI_ shows thermal stability up to 60°C (Lee *et al*. 2015), alternations in the tertiary structure of sHsp20_GI_ upon temperature or other stress challenges has not been characterized yet.

Conventionally, heat shock proteins including sHsps are specifically transcriptionally induced at elevated temperature under the transcriptional control of the heat shock sigma factor 32 (RpoH) (Yura and Nakahigashi 1999). This tight control extends to the posttranscriptional level as the ROSE element of repression of heat shock gene expression in the 5′-untranslated region of sHsps inhibits expression at temperatures below 30 °C (Kortmann and Narberhaus 2012; Krajewski, Nagel and Narberhaus 2013). Of note, however, sHsp20_GI_, as other TLPQC gene products (see below, (Lee *et al*. 2018)), shows an unconventional expression pattern. sHsp20_GI_ is produced between 20°C and 42°C from mid-logarithmic phase with maximum expression in stationary phase in both minimal and rich medium (Lee *et al*. 2015). Oxidative stress can further enhance production of sHsp20_GI_ (Lee *et al*. 2015). Of note, sHsp20_GI_ is one of the most highly expressed proteins of *P. aeruginosa* clone C strains under standard growth conditions (Sriramulu, Nimtz and Römling 2005).

**Figure 10.**
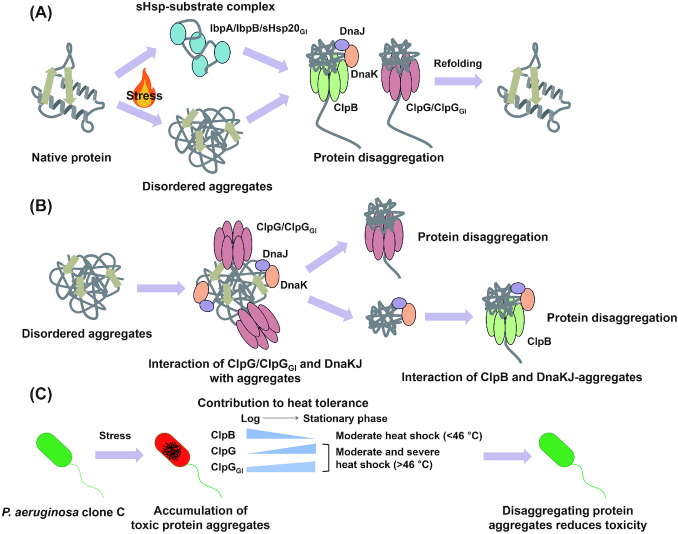
Processing of stress-induced protein aggregates by ClpG and ClpB chaperones. **(A)** Various stresses, such as elevated temperature, can lead to disordered aggregation of native proteins. sHsps form a complex with denatured proteins to prevent irreversible protein aggregation as well as to facilitate the disaggregating and refolding by ClpB-DnaKJ/ClpG chaperones. **(B)** ClpG, ClpG_GI_ and the DnaK chaperone directly bind disordered aggregates. ClpG and ClpG_GI_ bind to the disordered aggregates with their N-terminal domain to subsequently perform disaggregation. Although the DnaK/DnaJ/GrpE system can dissolve aggregates to some extent, aggregate-loaded DnaK/DnaJ/GrpE associates with the M-domain of the disaggregase ClpB to activate ATP-ase activity and disaggregation. **(C)** Aggregation of proteins, which have a vital role in the cell, has a detrimental effect on cell physiology. The ClpB-DnaKJ disaggregating chaperone complex functions efficiently at moderate heat shock condition, which is up to 46°C, in the logarithmic phase. ClpG and ClpG_GI_ mainly work in the stationary phase of growth and their potent threading power allows them to disaggregate proteins formed under severe heat shock conditions (Lee *et al*. 2018). Unlike ClpG, ClpG_GI_ contributes to heat tolerance in the logarithmic growth phase and backs up the ClpB-DnaKJ system.

Conventionally, sHsp20s are holding chaperones, which can prevent the thermal aggregation of client proteins (Fig. 10). Such an activity was also demonstrated for sHsp20_GI_ using the model substrate citrate synthase (Lee *et al*. 2015). Furthermore, as for other sHsps, deletion of *shsp20_GI_* slightly, but significantly reduces heat tolerance. Redundancy in thermotolerance functionality is demonstrated upon co-deletion of the *P. aeruginosa* gene *ibpA* coding for the core genome sHsp20. In *E. coli*, the sHsps IbpA and IbpB work cooperatively with disaggregating and refolding chaperones such as ClpB, DnaK and GroELS (Zolkiewski 1999; Mogk *et al*. 2003; Mogk, Ruger-Herreros and Bukau 2019). Upon thermo and other stress conditions, sHsps create a reservoir of client proteins to prevent irreversible aggregation, which provides an amenable protein state for disaggregation and subsequent refolding (Mogk *et al*. 2003; Mogk, Ruger-Herreros and Bukau 2019). Although the operon context of *shsp20_GI_* in combination with *clpG_GI_* encoding the disaggregating chaperone suggests cooperativity, such a functionality has not yet been examined.

### The stand-alone disaggregase ClpG/ClpG_GI_

Together with the Mer-like transcriptional regulator *dna* and *shsp20_GI_*, *clpG_GI_* composes the core unit of any TLPQC locus. As other Hsp100 family members, ClpG_GI_ contains two distinct AAA + domains with a M-domain integrated into the first AAA + domain to form a hexameric structure (Lee *et al*. 2016, 2018). ClpG_GI_ shows the highest amino acid homology to the well characterized class I AAA + chaperones ClpC and the disaggregase ClpB, but has distinctively longer N- and C-terminal domains and a unique M-domain sequence. Although a species specific protease interaction motif cannot be excluded (Miller, Chaudhary and Marsee 2018), VGF protease interaction motif present in *Bacillus subtilis* ClpC, which couples protein disaggregation with proteolytic digest (Trentini *et al*. 2016), is missing. Accordingly, ClpG_GI_ has been characterized as a disaggregating chaperone with distinct features compared to the ClpB-DnaK/DnaJ/GrpE bichaperone system. Most characteristic, in contrast to ClpB, purified ClpG_GI_ is able to potently disaggregate client proteins without requiring the assistance of the accessory DnaK/DnaJ/GrpE helper chaperone system (Fig. 9; (Lee *et al*. 2018)). Consistent with *in vitro* results, *clpG_GI_* expression confers a heat tolerant phenotype and solubilizes the heat-induced protein aggregates in a *clpB* or *dnaK* deletion background.

The molecular basis of these unique features of ClpG_GI_ is based on the high basal ATPase activity and the extended N-terminal region that directly binds the substrate (Lee *et al*. 2018). These unique biochemical features of ClpG_GI_ have two consequences. First, the high ATPase activity of ClpG_GI_ calls for a repressive type of tight physiological regulation of ClpG disaggregases besides activation by aggregate loaded DnaK of the disaggregation activity of Clp_B_*in vivo*. Indeed, we have observed high molecular weight structures of ClpG_GI_ (Lee, Curth, Carroni and Römling, unpublished data) that probably represent an inactive state as it has been observed in other systems (Carroni *et al*. 2017).

Second, the feed-forward loop of the heat shock response regulon is disrupted as transcription of *clpG_GI_* is independent of sigma 32 and, at the same time, direct substrate binding uncouples protein disaggregation by ClpG_GI_ from activation by the aggregate-loaded anti-sigma factor DnaK/DnaJ/GrpE. The lack of this regulatory circuit suggests that ClpG_GI_ mainly disassembles protein aggregates beyond saturation of the capacity of the DnaK/DnaJ/GrpE system (as DnaK and ClpG_GI_ compete for a certain type of aggregates (Katikaridis*et al*.^2019^)), beyond recognition by DnaK/DnaJ/GrpE or created by severe stress conditions alternative to elevated temperature that do not induce the heat shock regulon.

Furthermore, we have found that *P. aeruginosa* harbours a *clpG_GI_* homologue in the core genome. ClpG shows similar features as ClpG_GI_ such as the high ATPase activity and substrate binding by the extended N-terminal domain suggesting that those two features are hallmarks of the ClpG family within the Hsp100 superfamily (Lee *et al*. 2016, 2018). Nevertheless, dissection of the function of the extended N-terminal domain of ClpG/ClpG_GI_ showed that the extended part of the N-terminal domain (N2) is required for the disaggregation activity. Despite high homology, upon deletion, distinct functionality of the N2 domain between ClpG and ClpG_GI_ is observed. While the N2 domain of ClpG was required for substrate binding, the N2 domain of ClpG_GI_ dramatically repressed the ATPase activity (Lee *et al*.^2018^).

*Pseudomonas aeruginosa* is the predominant human pathogen in the *Pseudomonas* genus, and the only species harbouring a monocistronic *clpG* gene in the core genome. Together with an altered GC content of the ORF, *clpG* has been acquired by horizontal gene transfer upon speciation of *P. aeruginosa*. Why does *P. aeruginosa* redundantly possess both a ClpB and a ClpG disaggregating chaperone? Indeed, while *clpB* is active in the logarithmic phase of growth, *clpG* regulated by the oxygen-sensing transcriptional regulator Anr and under direct or indirect control of the PhoP/PhoQ two-component system mainly shows activity in the stationary phase of growth ((Gooderham *et al*. 2009; Trunk *et al*. 2010; Babin *et al*. 2016); (Fig. 9C)). Of note, core genome *clpG* seems to encode a multifunctional gene product deeply involved in *P. aeruginosa* physiology as it is also required for dispersion-responsive biofilm formation in *P. aeruginosa* (Petrova and Sauer 2012), is produced in elevated amounts in human urinary catheter biofilms (Lassek *et al*. 2015) and has been found to be required for virulence in a rat model of chronic infection in a transposon screen (Potvin *et al*. 2003). Of note, as *clpG* is consistently upregulated under low oxygen and anaerobiosis with expression induced by nitrate (Filiatrault *et al*. 2005; Alvarez-Ortega and Harwood 2007) cumulatively those data suggest that *clpG* provides an advantage to *P. aeruginosa* upon oxygen limitation. And why do *P. aeruginosa* clone C strains redundantly possess even two ClpG-like disaggregating chaperones? Heat shock sensitivity experiments with mutants have shown that *clpG_GI_*has a major role in the logarithmic growth phase where it backs up mainly *clpB*, while it backs up mainly *clpG* in the stationary phase of growth (Fig. 10C; (Lee, Kim and Bardwell 2018)). Whether *clpG_G_*_I_ also has a backup function during oxygen limiting conditions, needs to be further demonstrated.

How do ClpB and ClpGs differently contribute to bacterial protein homeostasis? The production of ClpB and ClpGs is distinct suggesting that the timing and regulation of expression contributes to the differential role of these diaggregases. ClpB expression is neglectable at 37°C, but induced upon heat shock by RpoH (Kitagawa *et al*. 1991; Lee *et al*.^2018^). On the other hand, both ClpGs are highly and constitutively expressed in the stationary phase of growth from 20°C to 42°C. Furthermore, ClpG confers superior heat tolerance at higher temperature compared to ClpB (Katikaridis *et al*. 2019). Consistently, *in vitro* ClpG exhibits robust activity towards diverse protein aggregates formed at elevated temperature whereby the ClpB system shows poor activity. These results suggest that ClpG exerts potent disaggregation to tight aggregates formed during severe thermal stress.

ClpB is widespread in bacteria, but the occurrence of ClpG class proteins is restricted mainly to single strains within a species or to extremely heat-tolerant strains (Mercer *et al*. 2015). Why is ClpG not as relevant as ClpB despite superior biochemical characteristics? As discussed above, the ClpB/DnaK system works as a very efficient dissaggreage at moderate heat shock condition up to 46°C (Katikaridis *et al*. 2019) and is tightly integrated into the heat shock regulon with respect to production, functionality and regulation. Thus, it can be argued that sudden elevation of temperature to sublethal and lethal temperatures seem to be rare. However, microorganisms are exposed to elevated temperature stress (57–68°C) during food such as cheese and milk production and sterilization of medical devices. These environmental settings lead to selection of ClpG_GI_-harboring *E. coli* strains and 2/3 of *Klebsiella pneumoniae* strains from clinical environment harbour ClpG (ClpK) (Bojer *et al*. 2010; Jorgensen *et al*. 2016). Furthermore, in *E. coli*, the TLPQC island promotes also tolerance to other type of stresses such as pressure and oxidative stress (Li *et al*. 2020; Wang *et al*. 2020).

Within the *Pseudomonas* genus, the human pathogen *P. aeruginosa* is the only species with a growth temperature up to 42°C, which implies a potential role of *clpG* in bacterial virulence, antibiotic resistance and host–microbe interaction. Additionally, revealing physiological substrates of ClpG will give us insight to understand more precisely the role of ClpG in the species *P. aeruginosa*. Regulatory mechanism to control the constitutive ATPase activity and cooperativity with the functionally associated sHsp20_GI_ still remains to be investigated.

### The membrane-bound protease FtsH

The multifunctional inner membrane anchored protease FtsH (Ito and Akiyama 2005) is ubiquituously present in Gram-negative and Gram-positive bacteria, archeae, chloroplasts and mitochondria. FtsH is a multidomain protein composed of an N-terminal periplasmic region flanked by transmembrane helices, a distinct AAA domain with a characteristic second region of homology and a C-terminal M41 proteinase domain. Aided by the transmembrane helices and the AAA domains FtsH assembles into a hexamer which unfolds and translocates substrates into the proteolytic chamber at the C-terminus for hydrolysis into 5–28 amino acid long peptides (Ito and Akiyama 2005). The catalytic site of the C-terminal M41 proteinase contains a conserved HEXXH motif coordinated Zn^2+−^ion (Ito and Akiyama 2005).

Functionality and regulation of the bacterial FtsH protease has been mainly explored in the model bacterium *Escherichia coli* K-12. The hexameric FtsH is part of a multi-protein complex and associates with modulator proteins such as HflC and HflK, with prohibitins as eukaryotic homologues, to regulate proteolytic activity towards membrane proteins (Kihara, Akiyama and Ito 1996). Major substrates of FtsH are (i) out-of-context membrane proteins, (ii) truncated and misfolded *ssrA* tagged proteins and (iii) proteins with partially unfolded loops. As one of the physiologically most relevant substrates FtsH mediates the efficient degradation of the heat shock responsive sigma factor RpoH at non-stress temperature (Fig. 10; (Tomoyasu *et al*. 1995)). Another key physiological feature of FtsH is regulation of the balance between LPS and phospholipids with the UDP-3-O-(R-3-hydroxymyristoyl)-N-acetylglucosamine deacetylase LpxC, a key enzyme involved in biosynthesis of the lipid A anchor of lipopolysaccharide (Ogura *et al*. 1999), as a major substrate. In addition, FtsH is involved e.g. in the decision between lysis and lysogeny upon bacteriophage infection by degrading CII and CIII and the regulation of basic energy and secretion processes by degradation of the subunit alpha of the F_1_F_0_ ATP synthase complex and the type 2 secretion system translocon protein SecY (Ito and Akiyama 2005).

Despite intensive investigations in *E. coli*, functionality of *ftsH* in *P. aeruginosa* is less well characterized. Of note, *ftsH* has a broad effect on physiology and metabolism as *ftsH* encoded on the core genome in *P. aeruginosa* clone C strains is required for the optimal growth of the organism in rich and defined medium and intrinsic resistance as well as tolerance of biofilms against clinically relevant aminoglycosides (Hinz *et al*. 2011; Kamal *et al*. 2019). Furthermore, *ftsH* is involved in a multitude of phenotypes from promotion of motility and biofilm formation to tolerance against hypochlorous acid and the production of secondary metabolites such as pyoverdine, phenazines and the *P. aeruginosa* quinolone signal (PQS) molecules (Kamal *et al*. 2019). Upregulated production in the Australien epidemic strain AES-1R compared with PAO and PA14 indicates a role of FtsH in facilitating early infection and transmission (Hare *et al*. 2012). Recent analysis also showed that FtsH is one of the few gene products that aids the survival of *P. aeruginosa* under both prolonged carbon and oxygen starvation in interplay with other proteases (Basta, Bergkessel and Newman 2017; Basta *et al*. 2020) Whether the multitude of phenotypes affected by FtsH requires the disaggregation and proteolytic activity and/or one of the alternative functions of FtsH, such as its reported chaperone or translocation activity (Schumann 1999; Chauleau *et al*. 2011), has not yet been fully sorted out.

In clone C strains, *ftsH2*, a xenologue of core genome *ftsH1*, is encoded on TLPQC-1 downstream of *clpG_GI_*. Of note, *ftsH2* backs up above described phenotypes in the absence of the *ftsH1* core genome copy. Nevertheless, FtsH2 on TLPQC-1 is constitutively produced throughout the growth phase with maximum level in the late stationary growth phase (Kamal *et al*. 2019) suggesting distinct mechanisms of regulation for *ftsH2*. The neglectible production of FtsH1 in late stationary phase suggests a unique role of FtsH2 in stationary phase with specific substrates (Kamal *et al*. 2019). In addition, experimental indications for hetero oligomer suggest broadened substrate specificity or proteolytic activity upon co-expression of FtsH1 and FtsH2 (Kamal *et al*. 2019). In conclusion, FtsH is involved in various phenotypes, important for bacterial fitness and adaptation in both environmental and clinical niches.

The heat shock sigma factor Sigma 32 is a major substrate of core genome FtsH1 indicating that in *P. aeruginosa* FtsH1 is a major regulator of the heat shock response. Similar as with the phenotypes, at most, FtsH2 backs up degradation of Sigma 32 in the absence of FtsH1. Pull-down of cross-linked tagged FtsH1 and FtsH2 protein complexes identified a number of interacting proteins several of them consistently substrates of FtsH in *E. coli*. Phenotypes and degradation studies confirmed PhzC, which channels precurser compounds into the synthesis of the phenazine pyocyanin, to be a substrate of FtsH1 (Kamal *et al*. 2019).

### Description of additional TLPQC-1 core gene products

**Figure 11.**
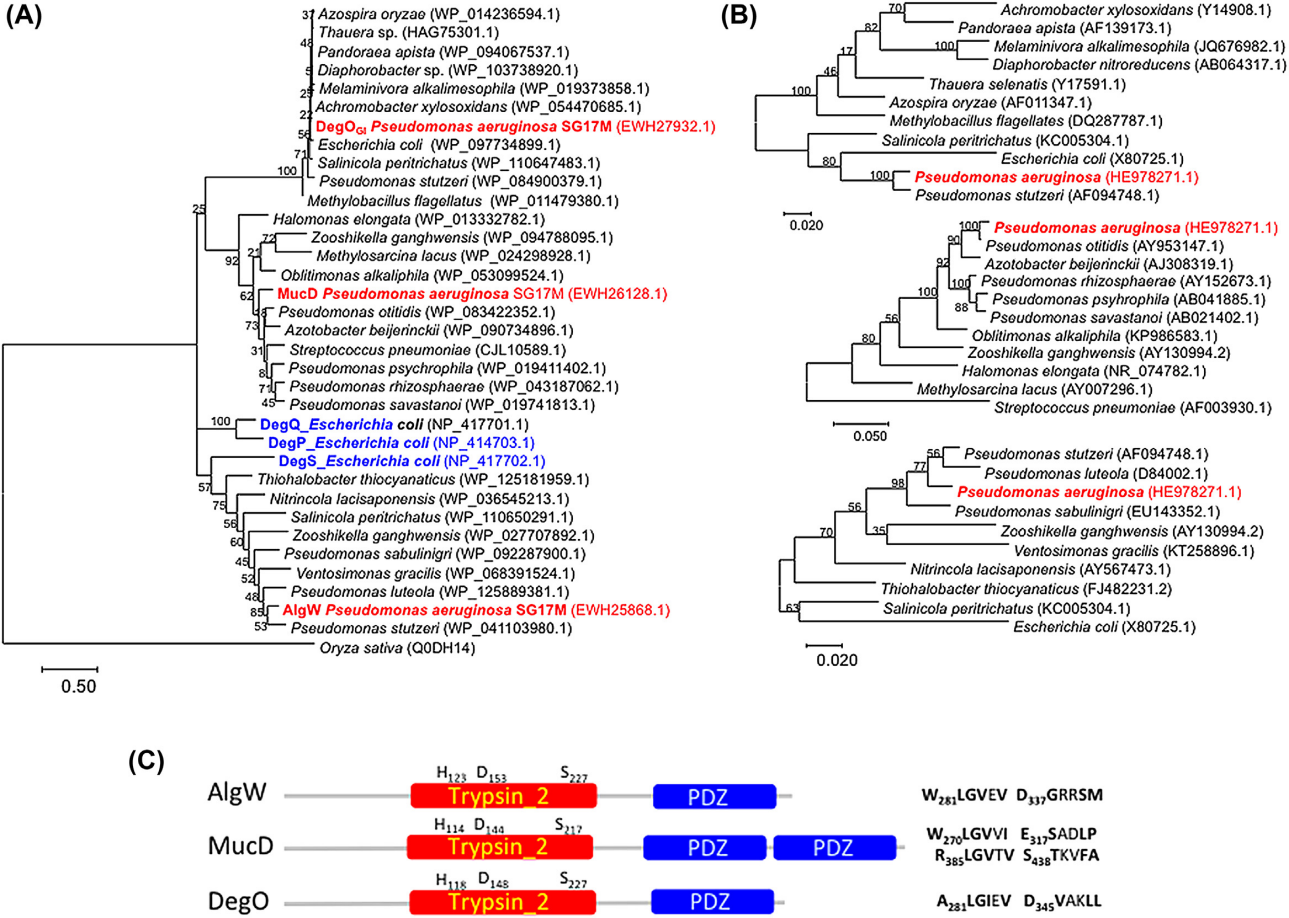
Phylogenetic analysis showing the position of the three HtrA homologues of *P. aeruginosa* clone C. **(A)** Phylogenetic analysis of HtrA/Deg proteases of *Pseudomonas aeruginosa* SG17M with closest homologues from different taxonomic groups of bacteria for each of the proteins. *P. aeruginosa* possesses the two HtrA/Deg homologues AlgW (DegS) and MucD (DegT), while *P. aeruginosa* clone C possesses DegO_GI_ as an additional HtrA/Deg homologue. Proteins DegS, DegP and DegQ of *E. coli* K-12 were included as reference. DegP of *Oryza sativa* is included as outgroup. **(B)** A corresponding 16S rRNA phylogenetic tree was constructed for each of the HtsA/Deg subgroups. Branch lengths correspond to substitutions per site, bootstrap values are indicated in %. The sequence accession numbers are shown in parenthesis. Maximum likelihood method from MEGA 7 program was used for phylogenetic analysis. **(C)** Domain structure of the three HtrA/Deg homologues of *P. aeruginosa* clone C. Trypsin_2 is the trypsin-like peptidase domain with conserved residues H/D/S involved in catalysis indicated; PDZ indicates the PDZ_serine_protease domain associated domain involved in substrate binding. Predicted protein binding sites (https://blast-ncbi-nlm-nih-gov.proxy.kib.ki.se/Blast.cgi) are indicated.

Besides the core operon *dna-shsp20_GI-_clpG_GI_*and *ftsH*, additional horizontally transferred genes are encoded on TLPQC-1. The gene products show consistency in physiological function,which indicates involvement in stress response, primarily towards high temperature, and antimicrobial treatment. Many of those genes are xenologues of conserved core genome genes, and consequently possess a distant homologue on the core genome. For example, three proteases, with identified core genome homologues required for high temperature physiology, are encoded on the island; FtsH (described above), DegO_GI_ (a homologue of *E. coli* DegQ/DegP/DegS (HtrA (high temperature requirement A)); Fig. 11) and HtpX. Besides TLPQC-1 located DegO_GI,_ core genome DegS (AlgW) and DegT (MucD) are present in *P. aeruginosa* (Fig. 11A and B). The three proteins have a similar domain structure with a N-terminal trypsin_2 peptidase domain and one or two C-terminal PDZ substrate binding domains (Fig. 11C). Both, AlgW and MucD, are involved in the repression of alginate production, the characteristic exopolysaccharide of biofilm-forming *P. aeruginosa* CF lung isolates (Boucher *et al*. 1996). Of note, close similarity to the functionally well-characterized protease and chaperone DegP (which includes closely related DegQ and DegS serine peptidase homologues) in *E. coli* is restricted to the 42.6% identitical DegS/AlgW which seems to be a member of the DegS subfamily. In contrast, DegO_GI_ and DegT belong to distinct subfamilies (Fig. 11A and B). HtpX, another membrane-bound Zn^2+^ dependent peptidase, shows overlapping functionality with FtsH in *E. coli* (Yoshitani, Hizukuri and Akiyama 2019).

Another example of a gene product, present on TLPQC-1, and conserved throughout the phylogenetic tree is the ubiquitous redox protein thioredoxin. The thioredoxin copy on TLPQC-1 is a distant homologue of thioredoxin 2 of *E. coli* and coexists with the core genome *E. coli* homologues of thioredoxin 1 and thioredoxin 2. Introduction of xenologues by horizontally transferred elements have been observed previously in *P. aeruginosa* (Liang *et al*. 2001). A prominent example of functionality diversification in bacteria is the occurrence and task distribution of four FtsH proteases in the cyanobacterium *Synechocystis* sp. PCC 6803 (Mann *et al*. 2000; Zhang *et al*. 2007). Furthermore, many, including the above mentioned TLPQC-1 encoded gene products are conserved not only in bacteria, but throughout the phylogenetic tree in archaea, mitochondria and chloroplasts suggesting that those distinct functionalities of protein quality control have a global impact on persistence and survival of organisms.

### Concluding remarks

The species *P. aeruginosa* thrives in various habitats facing highly diverse environmental conditions. While early highly discriminatory molecular typing approaches defined the epidemic population structure of *P. aeruginosa* characterized by frequent recombination with few highly abundant clones thriving in various habitats, whole genome sequencing opened up to trace the evolution of individual isolates within a clonal complex. As highly successful clones are characterized by matching population-wide variability of accessory genetic elements, potentially a combination of core genome features and strain variability contributes to their success. The horizontal acquisition of a common gene cluster encoding protein homeostasis elements is believed to be a factor that contributes to clone C persistence in environmental and clinical niches. As not all successful *P. aeruginosa* clones harbor this genomic island, the question whether there are common genetic and phenotypic traits that characterize successful clones still remains open. Whether, and, if so, how invididual strains of successful clones coexist in a microniche is another open question.

## Supplementary Material

fuaa029_Supplemental_File
