# Supplementary material for: Why? – Successful Pseudomonas aeruginosa clones with a focus on clone C
Source: FEMS Microbiol Rev. 2020 Sep 29;44(6):740–62. doi: 10.1093/femsre/fuaa029 (PMC7685784; doi:10.1093/femsre/fuaa029)
Supplement: fuaa029_Supplemental_File [file fuaa029_supplemental_file.docx]

| **Table S1: Numbers of strain-specific genes detected in strains of a clone C panel** | | | | | | |
| --- | --- | --- | --- | --- | --- | --- |
|  |  |  |  |  |  |  |
| **isolate** | **number of strain-specific genes** | **thereof with ortholog from *Pseudomonas aeruginosa*** | **thereof with ortholog from other Pseudomonads** | **thereof with ortholog from other γ-proteobacteria** | **thereof with ortholog from 'honorable Pseudomonads'** | **thereof with ortholog from other species** |
| 10SA1 | 403 | 104 | 124 | 171 | 0 | 4 |
| 12_F8 | 96 | 61 | 16 | 4 | 4 | 11 |
| 12_SB1 | 216 | 108 | 36 | 22 | 9 | 41 |
| 17 | 79 | 61 | 4 | 2 | 6 | 6 |
| 28PA3 | 243 | 88 | 142 | 5 | 1 | 7 |
| 36 | 109 | 70 | 24 | 3 | 1 | 11 |
| 69 | 70 | 49 | 6 | 6 | 0 | 9 |
| 84 | 248 | 97 | 70 | 20 | 6 | 55 |
| 87 | 128 | 71 | 28 | 8 | 1 | 21 |
| 97 | 89 | 78 | 5 | 0 | 1 | 5 |
| 110 | 380 | 124 | 68 | 83 | 4 | 101 |
| 110_D4 | 156 | 98 | 36 | 8 | 1 | 13 |
| 115 | 86 | 59 | 18 | 1 | 0 | 8 |
| 124 | 64 | 53 | 0 | 0 | 0 | 11 |
| 142 | 128 | 80 | 29 | 6 | 0 | 13 |
| 159 | 227 | 120 | 71 | 20 | 1 | 15 |
| 179 | 64 | 51 | 4 | 1 | 0 | 8 |
| 214 | 104 | 65 | 25 | 6 | 1 | 7 |
| 237 | 48 | 41 | 1 | 0 | 0 | 6 |
| 240 | 16 | 8 | 3 | 1 | 0 | 4 |
| 343 | 77 | 60 | 5 | 2 | 2 | 7 |
| 426 | 89 | 65 | 8 | 4 | 1 | 11 |
| 584 | 63 | 53 | 4 | 1 | 0 | 5 |
| 642 | 110 | 60 | 25 | 5 | 3 | 17 |
| 702_D3 | 86 | 58 | 10 | 1 | 0 | 17 |
| 747 | 165 | 96 | 33 | 17 | 3 | 16 |
| 800 | 57 | 50 | 1 | 0 | 0 | 6 |
| 902_D1 | 214 | 106 | 28 | 69 | 1 | 10 |
| AL962 | 197 | 83 | 77 | 12 | 6 | 19 |
| C17 | 140 | 78 | 23 | 14 | 0 | 25 |
| CF2 | 350 | 213 | 88 | 26 | 3 | 20 |
| CF3 | 189 | 115 | 58 | 4 | 1 | 11 |
| CF5 | 177 | 110 | 53 | 6 | 1 | 7 |
| CF7 | 91 | 71 | 11 | 2 | 0 | 7 |
| CF8 | 193 | 124 | 50 | 5 | 2 | 12 |
| CF10 | 121 | 83 | 20 | 7 | 3 | 8 |
| CF11 | 115 | 88 | 14 | 4 | 3 | 6 |
| K5 | 56 | 45 | 2 | 2 | 0 | 7 |
| K11 | 96 | 74 | 12 | 2 | 1 | 7 |
| K13 | 100 | 68 | 12 | 1 | 11 | 8 |
| K15 | 57 | 44 | 1 | 2 | 0 | 10 |
| K16 | 155 | 86 | 21 | 23 | 4 | 21 |
| K17 | 516 | 244 | 193 | 31 | 11 | 37 |
| K18 | 188 | 127 | 35 | 6 | 4 | 16 |
| K19 | 199 | 89 | 82 | 11 | 3 | 14 |
| K20 | 104 | 63 | 13 | 10 | 3 | 15 |
| NN2 | 11 | 7 | 1 | 1 | 0 | 2 |
| PJC | 70 | 57 | 4 | 1 | 0 | 8 |
| SG1 | 1 | 0 | 0 | 1 | 0 | 0 |
| SG17M | 90 | 72 | 5 | 1 | 1 | 11 |
| SG50M | 59 | 47 | 3 | 1 | 0 | 8 |
| SS30 | 103 | 87 | 3 | 5 | 0 | 8 |
| W5Aug28 | 90 | 67 | 7 | 2 | 2 | 12 |
| WI1 | 79 | 65 | 4 | 1 | 0 | 9 |
| WU2 | 126 | 108 | 4 | 7 | 0 | 7 |
|  |  |  |  |  |  |  |
| median | 104 | 71 | 14 | 4 | 1 | 10 |
| average | 136,1454545 | 79,07272727 | 29,45454545 | 11,89090909 | 1,909090909 | 13,81818182 |
|  |  |  |  |  |  |  |
| total | 7488 | 4349 | 1620 | 654 | 105 | 760 |
| Reference: Fischer S, Klockgether J, Moran Losada P*, et al.* (2016) Intraclonal genome diversity of the major *Pseudomonas aeruginosa* clones C and PA14. *Environ Microbiol Rep* **8**: 227-234 | | | | | | |
